# Triboelectric Nanogenerators for Self-Powered Degradation of Chemical Pollutants

**DOI:** 10.1021/acsomega.4c07889

**Published:** 2024-12-26

**Authors:** Md Mazbah Uddin, Tanvir Mahady Dip, Shariful Islam Tushar, Abdullah Sayam, Habibur Rahman Anik, Md. Reasat Aktar Arin, Amit Talukder, Suraj Sharma

**Affiliations:** † Department of Textiles, Merchandising, and Interiors, University of Georgia, Athens, Georgia 30602, United States; ‡ Department of Materials, University of Manchester, Manchester M13 9PL, United Kingdom; § Department of Yarn Engineering, Bangladesh University of Textiles, Dhaka 1208, Bangladesh; ∥ Department of Design and Merchandising, Oklahoma State University, Stillwater, Oklahoma 74078, United States; ⊥ Department of Textile Engineering, Ahsanullah University of Science and Technology, Dhaka 1208, Bangladesh; # Department of Apparel Engineering, Bangladesh University of Textiles, Dhaka 1208, Bangladesh; g Department of Chemistry & Chemical and Biomedical Engineering, University of New Haven, New Haven, Connecticut 30605, United States; h Department of Fabric Engineering, Bangladesh University of Textiles, Dhaka 1208, Bangladesh

## Abstract

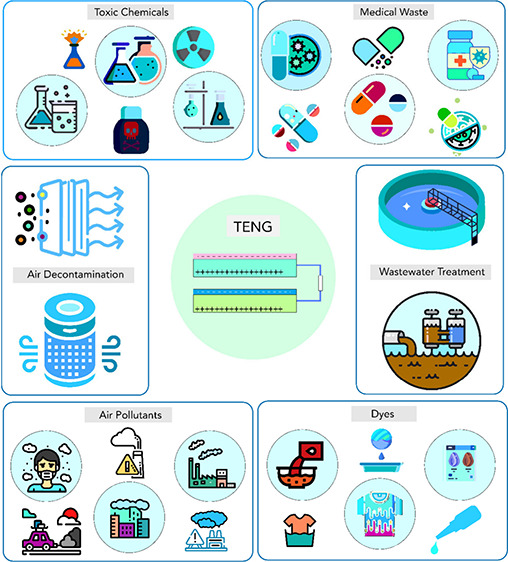

Environmental and human health is severely threatened by wastewater and air pollution, which contain a broad spectrum of organic and inorganic pollutants. Organic contaminants include dyes, volatile organic compounds (VOCs), medical waste, antibiotics, pesticides, and chemical warfare agents. Inorganic gases such as CO_2_, SO_2_, and NO_*x*_ are commonly found in polluted water and air. Traditional methods for pollutant removal, such as oxidation, physicochemical techniques, biotreatment, and enzymatic decomposition, often prove to be inefficient, costly, or energy-intensive. Contemporary solutions like nanofiber-based filters, activated carbon, and plant biomass also face challenges such as generating secondary contaminants and being time-consuming. In this context, triboelectric nanogenerators (TENGs) are emerging as promising alternatives. These devices harvest ambient mechanical energy and convert it to electrical energy, enabling the self-powered degradation of chemical pollutants. This Review summarizes recent progress and challenges in using TENGs as self-powered electrochemical systems (SPECs) for pollutant degradation via photocatalysis or electrocatalysis. The working principles of TENGs are discussed, focusing on their structural flexibility, operational modes, and ability to capture energy from low-frequency mechanical stimuli. The Review concludes with perspectives and suggestions for future research in this field, hoping to inspire further interest and innovation in developing TENG-based SPECs, which represent sustainable and eco-friendly solutions for pollutant treatment.

## 1 Introduction

Rapid industrialization and urbanization have led to a large amount of wastewater and contaminated air being discharged into the environment without proper treatment.^1−3^ These wastes contain various organic and inorganic pollutants that can cause serious environmental problems and health hazards.^4−8^ In general, industries such as chemical plants, textile factories, dyeing and printing plants, pharmaceuticals, and cosmetics release harmful gases, chemicals, and dyes that pollute the environment and water bodies and harm the lives of plants, animals, and people.^9−11^ These issues are now more severe than ever due to rapid technological development and demographic expansion. Human health is constantly being threatened by atmospheric pollution from particulate matter (PM) and gaseous pollutants, such as volatile organic compounds (VOCs), SO_2_, and NO_*x*_ gases.^12,13^ For instance, PM in the lungs can exacerbate diseases, such as asthma and bronchitis. Long-term exposure to polluted air can give rise to respiratory diseases, lung cancer, and cardiopulmonary mortality.^14,15^ The continuous presence and rapid spread of VOCs, such as formaldehyde, can cause discomfort and pose a toxic risk to humans.^16−19^ Therefore, it is urgent to develop effective methods for removing these pollutants from wastewater and air to protect the environment and human health.

Different techniques have been proposed for pollutant treatment, such as advanced oxidation processes,^20,21^ physicochemical techniques,^22−25^ biological treatments,^26−28^ enzymatic decomposition,^29^ and electrochemical techniques.^30−32^ First, advanced oxidation processes are a group of chemical treatment procedures intended to break down pollutants using powerful oxidants such as hydroxyl radicals (•OH). They offer fast reaction rates but have disadvantages because they can generate secondary pollutants and frequently demand large amounts of energy. Second, a physicochemical process denotes the essential interactions and changes occurring at the interface of substances’ physical and chemical properties. Adsorption and filtration are physicochemical processes that alter substances’ physical characteristics and chemical structures. These methods are simple and economical, but they are effective only on pollutants and require further treatment stages. Third, biological treatment is a prominent technology that uses microbes to degrade organic pollutants into nonhazardous ones. Although they work better on biodegradable contaminants than nonbiodegradable ones, biological treatments use microbes to break down organic pollutants, offering an affordable and environmentally friendly option. Also, applying a pretreatment process to hydrolyze and dissolve compounds may improve the biological treatments. Fourthly, enzymatic decomposition is a precise, low-energy process that targets contaminants by breaking down complex organic chemicals into simpler molecules using enzymes. However, enzymes are sensitive to environmental factors. Finally, electrochemical techniques directly oxidize or reduce pollutants on electrode surfaces, offering high efficiency in treating wastewater containing several organic and inorganic compounds. They are safe and environmentally friendly but face high operational costs and electrode fouling challenges. Hence, these techniques have several limitations and drawbacks. Common challenges include poor performance efficiency, complex structures and operation procedures, high costs, the generation of secondary contaminants, time consumption, and limited applicability. Some more advanced PM removal techniques, like high-efficiency particulate air (HEPA) filters, nanofiber-based filters, activated carbon absorption, and plant species, have recently gained popularity.^33−35^ However, they are also not free of challenges. For instance, HEPA and nanofiber filters are ineffective against VOCs; activated carbon may produce secondary contaminants and has limited absorptivity, and removal by plants is time-consuming. Another popular technique, electrostatic absorption, can remove PM efficiently; nevertheless, it suffers from high energy requirements and ozone generation problems.^13,36^ The electrochemical approach is significantly more popular than other methods due to its numerous advantages. These include adaptability, simplicity, environmental friendliness, operation at low temperatures, absence of sludge formation, and superior effectiveness.^37−39^ However, it also requires a significant amount of energy, which further burdens the already depleting fossil energy resources and makes it economically inefficient.^2,40^

As an alternative attempt, problems associated with high energy requirements could be resolved by accessing green, sustainable, energy-saving, and low-cost methods to degrade or remove these hazardous chemicals and prevent further environmental and public health deterioration.^41,42^ In this regard, the utilization of often wasted or underutilized renewable energies, such as mechanical, thermal, solar, and biochemical, has advanced significantly during the past decade.^43−45^ Recently, mechanical energy harvesting nanogenerators (NGs), such as piezoelectric NGs (PENGs) and triboelectric NGs (TENGs), have emerged as promising technologies for generating electrical energy for low-power consuming electronic devices, self-powered sensors, micro/nanosystems, and electrochemistry.^46−50^ Furthermore, thermoelectric NGs (TEGs), pyroelectric NGs (P_*y*_NGs), solar cells (SCs), and biofuel cells (BFCs) can be applied independently or in combination to develop hybrid NGs (HNGs) that may utilize multiple energy sources individually or simultaneously.^45^ In most cases, harvesting performance depends on weather, day/night cycle, and physical locations, which might not always be conducive.^43,45,51−60^

Among NGs, TENGs have drawn the most attention as they are based on the principles of contact electrification and electrostatic induction, and they can produce high voltage and power output with simple designs and low-cost materials.^45,61−65^ Furthermore, TENGs can harvest low-amplitude mechanical energy from ambient sources such as light, wind, water waves, raindrops, and human movements.^66−69^ TENGs have great potential in powering micro/nanosystems, self-powered sensors, blue energy, and direct high-voltage power sources.^70−72^ Moreover, TENGs have also been applied to self-powered electrochemical systems (SPECs) for chemical synthesis, degradation, and detection of chemical compounds,^9,47,73−76^ heavy metal adsorption and removal,^77,78^ electrochemical processes,^79^ oxidation of hazardous gases,^13,80^ water splitting for fuel production,^57,81^ organic pollutant degradation,^10,82−84^ seawater desalination,^85^ and anticorrosion processes.^86^ By integrating TENGs with different catalysts and electrodes, SPECs can achieve photocatalysis or electrocatalysis of chemical pollutants without external power sources, thus providing a renewable, sustainable, and eco-friendly solution for pollutant treatment. With features like high energy conversion efficiency, inexpensive materials, easy construction, light weight, simple operation, and reusability, TENGs could provide a renewable pathway for wastewater treatment and ecological sanitation to prevent the loss of environmental and public health and reduce the burden on the global energy demand.^87−90^ Overall, TENGs include a novel method that captures ambient mechanical energy, including vibrations or fluid dynamics, rendering them self-sustaining. TENGs offer an environmentally safe alternative with negligible secondary pollutants and are especially appropriate for remote locations compared to the previously described technologies, including advanced oxidation processes, physicochemical procedures, biological treatments, enzymatic decomposition, and electrochemical techniques.

This Review provides a thorough examination of recent advancements in TENGs that have assisted SPECs in the degradation and elimination of chemical pollutants found in wastewater and air. The Review commences by introducing the working principles and modes of TENGs, as well as their integration with various catalysts and electrodes for applications in SPECs. The subsequent segment delves into the performance and mechanisms of TENG-based SPECs for the degradation of common organic pollutants, VOCs, and inorganic gases. The authors also delve into a critical analysis of the advantages and challenges associated with different modes of TENGs, various catalysts, and electrodes used for pollutant degradation. Lastly, the Review concludes with perspectives and recommendations for future research directions in this field. Through this Review, the authors attempted to spur more interest and innovation in developing TENG-based SPECs as sustainable and eco-friendly solutions for treating pollutants.

## 2 Different Operation Modes of TENGs

The underlying principle of TENGs is triboelectrification and the generation of static polarized charges due to electrostatic induction, which is related to Maxwell’s displacement current.^40,61,62,91^ It happens when materials with differing electron affinities come into contact due to pressing; opposing electrical charges are induced on their contact surfaces. As the materials move apart, a potential difference develops, which drives electrons across the electrodes on the materials’ noncontact surfaces to screen out the electric field.^46,61,92^ The potential difference on the electrodes is altered when these surfaces are brought back in the following step, and electrons will flow in the opposite direction; thus, a continuous AC output can be obtained by repeating such an operation cycle.^45,49,50^ TENGs are superior to conventional electromagnetic generators at low frequencies (<5 Hz). Because of their inherently high voltage and low current characteristics, they can be used as high-voltage power sources.^19,93−95^

**Figure 1:**
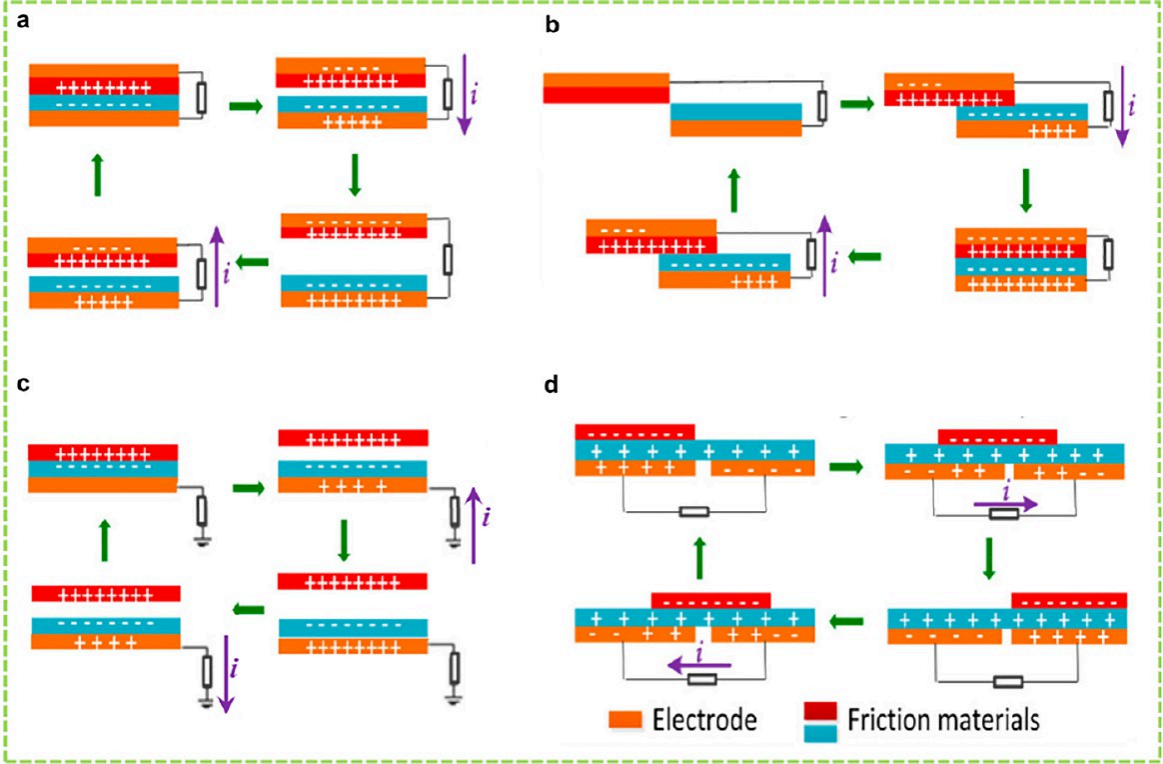
Schematic of the four working modes of TENGs. (a) CS mode, (b) LS mode, (c) SE mode, and (d) FS mode. Reprinted under the terms and conditions of the Creative Commons Attribution (CC BY) license (http://creativecommons.org/licenses/by/4.0/) from ref (105). Copyright 2018 MDPI.

Since Wang et al. discovered a TENG in 2012, researchers have introduced various methods of periodic contact separation among triboelectric layers to develop four fundamental modes of operation.^62,96^ The fundamentals of various working modes are essentially the same, and these modes are known as contact-separation mode (CS), linear sliding mode (LS), single electrode mode (SE), and free-standing (FS) triboelectric layer modes.^18,19,97−99^ The CS mode utilizes the relative perpendicular motion between the triboelectric layers at the contact interface, and the electrical output is determined by the appropriate separation distance between the triboelectric layers (Figure 1a).^61,100^ The CS-TENGs are simple in structure, have high durability, and can survive up to 1 000 000 cycles since surface abrasion is minimal. However, the overall packaging of the device can pose some difficulties due to the requirement for gap allowance between the triboelectric layers.^62,100^ The LS mode utilizes the relative lateral displacement between the triboelectric layers and in parallel to the contact interface (Figure 1b).^61^ This mode is useful for operation at a higher frequency and provides better energy conversion efficiency due to better contact between the triboelectric layers.^101^ Also, the charge density of these TENGs is much higher than the value of conventional TENGs.^102^ Compact packaging can be implemented; however, durability can be problematic for the LS mode due to high wear and tear.^100^ To address this issue, there is another variation of the lateral sliding mode designed explicitly for rotational motion, which is equivalent to connecting many LS mode TENGs in parallel. This mode relies on the relative rotation between the rotator and the stator. As the rolling element traverses the surface, it perpetually establishes and disrupts contact, resulting in recurrent charge transfer and separation cycles. The variation in the electric potential induces electron movement across an external circuit. This mode is beneficial because it minimizes material wear and tear and maximizes efficiency in transforming mechanical energy from rolling motion into electrical energy.^103,104,102^

TENGs operating in CS and LS modes feature electrically interconnected electrodes. In certain scenarios, these modes can effectively harvest energy from moving object. SE mode TENGs, designed to harvest energy from freely moving objects, have been introduced. Unlike other modes, they do not require an electrode to be attached to one of the triboelectric layers. Instead, a reference electrode is introduced between the attached electrode and an external load. (Figure 1c).^61,62^ This mode simplifies the structure of the TENGs, lends flexibility to the design, and can adapt contact separation and lateral sliding-based energy harvesting mechanisms. However, SE TENGs suffer from low energy output.^100^ The FS mode is somewhat based on the SE-TENGs; however, instead of the reference electrode, two symmetric electrodes are usually used. The asymmetric charge distribution between the electrodes due to the movement of the free-moving object is responsible for the electrical output (Figure 1d).^61^ This mode of mechanism allows the design of TENGs in rotational configurations and can harvest air and water flow conveniently.^62,100^ The FS mode endows TENGs with great robustness and high energy conversion efficiency and can be opted for sliding motions and grated structures proper for electrochemistry applications. The working mechanism of different modes is not addressed in detail, as these have been discussed well in the literature.^100,106^

The various operational modes available to TENGs have expanded their design and construction possibilities. TENGs can harness energy from sources such as air/wind, water waves, sound waves, and other mechanical vibrations, thus reducing the need for batteries and their associated upkeep and environmental impacts.

## 3 Mechanism of TENG-Assisted Degradation of Chemical Pollutants

Chemical pollutants can be classified into organic compounds, VOCs, and inorganic gases. Each chemical pollutant has different degradation mechanisms. Organic compounds, such as dyes, gummy materials, antibiotics, pesticides, urine contents, and chemical warfare agents and often originate from industrial, agricultural, and household applications. Because of their complicated molecular structures, they exist in the environment and can threaten human health. Photocatalysis and advanced oxidation processes are the best options for degrading these organic compounds. In the photocatalysis process, catalysts like TiO_2_ generate ROS that are activated by UV irradiation to degrade, and advanced oxidation processes intend to break down pollutants using powerful oxidants.^20,21,84^ Second, compounds of carbonyl and aromatic origin, such as formaldehyde, benzene, toluene, and xylene, are common VOCs, which have been classified as toxic chemical pollutants that readily vaporize at room temperature and are commonly released from industrial emissions, transportation, and various indoor and outdoor activities.^107,108^ The degradation methods for VOCs include the photocatalytic oxidation process, which transforms them into less dangerous substances; adsorption onto activated carbon or zeolites; and electrocatalysis, which decomposes VOCs on catalyst surfaces. Finally, inorganic gases, including CO, CO_2_, SO, NO_*x*_, H_2_S, and HF, are typically generated from industrial fumes, transport emissions, power plants, and natural incidents like volcanic eruptions.^109^ The methods for degrading inorganic gases encompass catalytic reduction, which transforms detrimental gases into innocuous products; electrocatalysis, which facilitates the oxidation or reduction of gases; and adsorption or absorption techniques that entrap gases on porous substrates or within liquid scrubbers.^110,111^ Each category of pollutant from these organic compounds, VOCs, and inorganic gases, which possess distinct characteristics, needs tailored degradation methods to successfully alleviate environmental and health repercussions

TENGs can generate a high electrostatic field by harnessing environmental vibrations, enabling the efficient removal of PM without producing ozone because electrostatic forces can attract and capture airborne particles. This electrostatic adsorption capability of TENGs makes this prominent. The ability of TENGs to convert low-frequency mechanical energy into a solid electrostatic field from environmental vibrations creates a charge on nearby PM particles. Then, PM particles experience a force pulling them toward oppositely charged collection surfaces, leading to attraction and trapping on these surfaces. Electrostatic precipitators can also perform this removal process, which requires high energy. Compared to that, TENGs use sustainable energy harvested from environmental vibrations without generating ozone as a byproduct.^112^ This is a significant advantage, as it prevents the creation of additional pollution while effectively treating the existing problem.^13,113,114^ Also, this electrostatic adsorption capability is significant for air pollution control, especially in filters, since PM particles are major indoor air pollutants linked to hazardous respiratory issues. However, TENGs have only electrostatic adsorption capability and cannot degrade VOCs, which is necessary for indoor air filtration. Limitations in VOC degradation exist because VOCs are gaseous molecules, and researchers use an oxidation process to break down the molecular structures of these gases.^115^ As TENGs work through electrostatic forces rather than chemical reactions, they cannot neutralize indoor air pollutants. Hence, photocatalysis^84^ and electrocatalysis^10^ degradation mechanisms are adopted in combination with TENGs to efficiently degrade harmful organic^80,82,116^ and inorganic^69,117^ chemical compounds into benign molecules. Released industrial effluents from chemical plants, textile and printing industries, cosmetics, pharmaceuticals, and agrowaste contain hazardous dyes, chemicals, and other organic/inorganic pollutants that severely pollute the environment, especially water bodies.^10,11,118^ Many of these pollutants are toxic, carcinogenic, and mutagenic and can cause organ failure.^14,15,119^ Therefore, research into treating industrial effluents/wastewater utilizing TENGs integrated SPECs made significant progress.^18,19,120,121^

Photocatalysis is a known technique for degrading aqueous and gaseous pollutants and can be integrated with TENGs easily for enhanced adsorption and chemical degradation by the TENGs.^122,123^ Under UV irradiation, the photocatalyst generates electron–hole pairs, which migrate to the surface of the photocatalyst and can induce redox reactions to degrade adsorbed materials by oxidation.^13,80^ Nevertheless, the efficacy of a photocatalyst for inducing a redox reaction at its surface is often hindered by the recombination of the generated free electron–hole pairs. Consequently, applying a strong electric field using TENGs can separate and help to migrate these electron–hole pairs to the photocatalyst surface more effectively, thus increasing photocatalyst quantum efficiency.^84,124^ In contact with oxygen and water, these electron–hole pairs can generate radical species, for example, superoxide (•O_2_^–^), hydrogen superoxide (HO_2_), hydroxyl ion (OH^–^), hydrogen peroxide (H_2_O_2_), and hydroxyl (•OH) radical species.^13,84^ The production of such radical species can directly or indirectly degrade atmospheric pollutants into benign H_2_O and CO_2_ molecules.^125−127^

Electrocatalysis is another effective technique to efficiently degrade chemical compounds into harmless molecules efficiently. However, an external power source is required to drive the redox reactions, which are energy-intensive, costly to operate, time-consuming, and necessitate expensive and complex instruments.^9,30,128^ Furthermore, the electro-Fenton (EF) process is used, where H_2_O_2_ is generated at the cathode, followed by its decomposition into •OH radicals via the catalytic effect of Fe^2+^ and regeneration of Fe^2+^ via the reduction of Fe^3+^ at the cathode. •OH is the reactive species with a higher oxidation potential that degrades organic pollutants. These •OH radicals can degrade organic compounds more efficiently in combination with TENGs.^129−132^

In the photocatalytic process, the catalyst is activated by UV irradiation to degrade the pollutants. TENGs can support this process by providing a continuous power supply to irradiate the UV lights. Again, in electrocatalytic degradation, the breakage of pollutants occurs when an electric field is applied, and TENGs could be a great source. Therefore, there is a need to combine photocatalytic and electrocatalytic degradation mechanisms with TENGs for pollutant degradation, leveraging TENGs’ energy-harvesting capabilities to increase photocatalyst quantum efficiency or generate reactive species essential for degrading organic and inorganic pollutants. This approach addresses the capture and degradation of chemical pollutants without external power sources, thus providing a renewable, sustainable, and eco-friendly solution for pollutant treatment.

TENGs are commonly built in FS or CS mode in rotating configurations for SPECs to capture the mechanical energy of air, water, and exhaust gas in addition to traditional stacked layer-based configurations in CS mode.^40,87,88,133^ Preinjected charges, nanopatterns, and buffer layers are some of the techniques that are also applied on the contact layer surfaces and are utilized to improve power conversion efficiency. In SPECs, electrical outputs from TENGs are usually transformed, rectified, and pulsed to have stable and efficient systems for the controlled and selective degradation of chemical compounds.

## 4 Degradation of Chemical Compounds by TENGs

Rapid industrialization has led to increased discharge of industrial effluents containing organic and inorganic pollutants harmful to the environment, such as dyes, coloring agents, and VOCs. One promising solution for sustainable degradation is devices assisted by TENGs. This section focuses specifically on the recent advancements in integrating TENGs to drive the efficient and sustainable degradation of a wide range of contaminants, including common organic compounds, VOCs, and inorganic pollutants.^134−137^ The goal is to provide a comprehensive overview of the potential of TENG-based systems in addressing environmental challenges.

### 4.1 Degradation of Organic Chemical Compounds

Organic compounds used in various industries can pose a threat to human health and the environment. Activities like fossil fuel combustion, pesticide use, and water purification can produce harmful organic pollutants, including persistent organic pollutants (POPs), which are particularly hazardous due to their toxicity and persistence in the environment. POPs have been found to pose severe health risks to humans and have detrimental effects on wildlife and aquatic mammals, highlighting the urgent need for sustainable solutions to mitigate their impact.^138,139^ Recent studies have demonstrated that many of these dangerous compounds can be degraded in SPECs by using TENGs as the power source. The current advancements in the chemical degradation of various organic components with the aid of TENGs are covered in the following sections.

#### 4.1.1 Organic Dyes

The widespread use of organic dyes and auxiliaries in various industries is causing serious environmental and human health implications. Azo dyes are of particular concern due to their production of carcinogenic aromatic amines and non-biodegradability. These pollutants persist in the environment, bioaccumulate in living organisms, and pose a significant risk. Researchers are working on finding sustainable solutions for dealing with such pollutants.^19,76,131^ Additionally, researchers worldwide are working on sustainable solutions to transform harmful dyes into harmless forms by using TENGs. This section provides insights into the current state of the application of TENGs in the degradation of organic dyes and their potential to address environmental challenges.

##### 4.1.1.1 Methyl Orange (MO)

**Figure 2:**
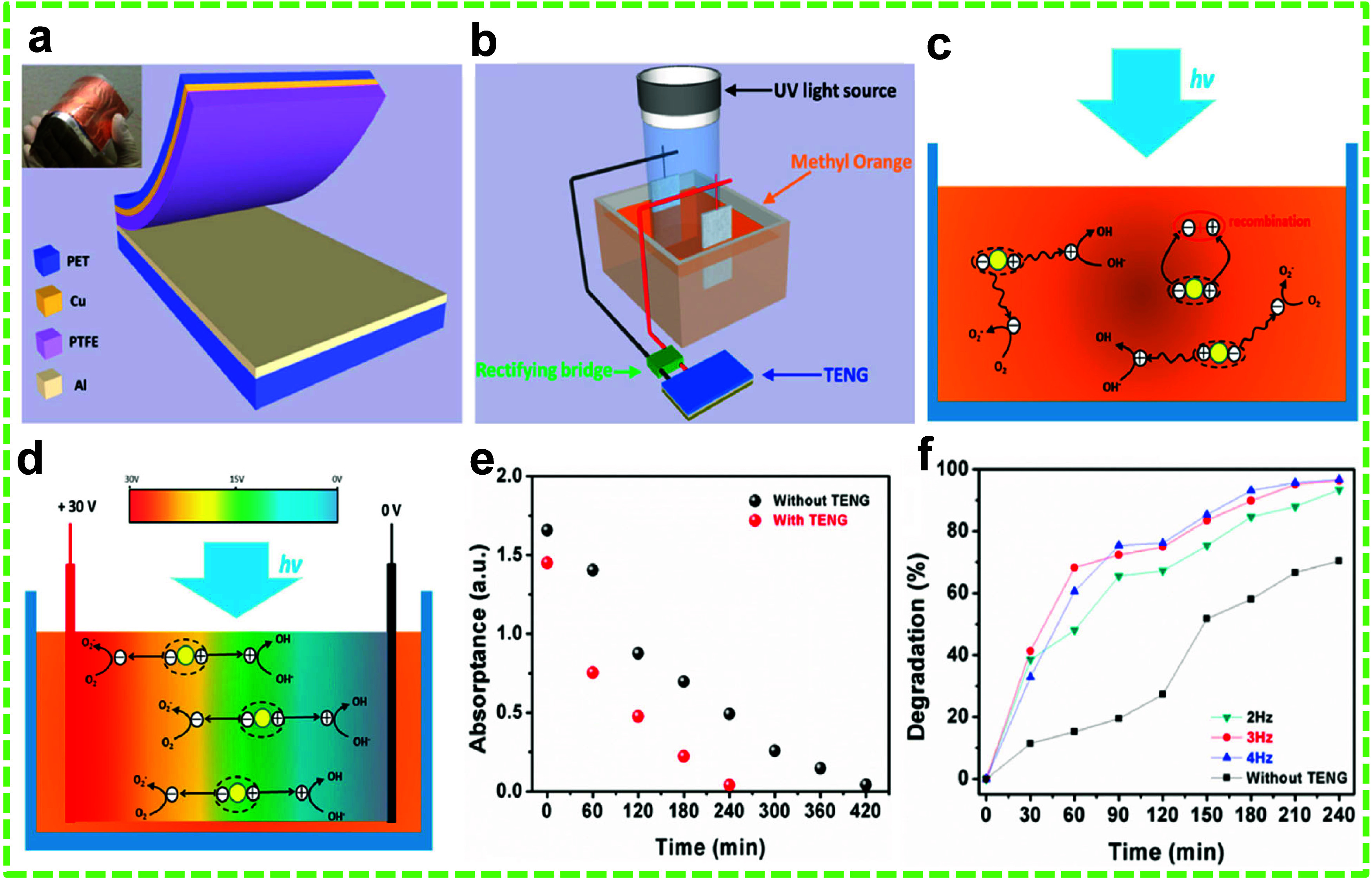
TENG-assisted PEC degradation of MO dye. (a) Schematic of the CS-TENG for TiO_2_ NP-enhanced PEC of MO. (b) Schematic of the setup for PEC of MO. Degradation mechanism of MO under UV light (c) in the absence and (d) in the presence of the TENG. (e) Absorption peak of MO at 464 nm against degradation time and (f) degradation percentage versus time with increasing frequency, both in the absence and presence of the TENG. Reprinted with permission from ref (84). Copyright 2013 IOP Science.

Photodegradation of organic dyes is a promising method, but its efficiency is often reduced due to the low charge separation and migration efficiency and higher recombination of electron–hole pairs in the photocatalysts. TiO_2_ is a well-studied photocatalyst for degrading various inorganic and organic compounds in both liquid and gaseous states.^140^ Su et al. studied the efficiency of TiO_2_ nanoparticles (NPs) in combination with a CS-TENG for photoelectrocatalytic (PEC) degradation of MO (Figure 2a and b).^84^ The TENG comprised a polytetrafluoroethylene (PTFE) film and aluminum (Al) foil as the friction layers, with Al foil also serving as one of the electrodes and copper (Cu) film serving as the other electrode. The objective was to generate a strong electric field to accelerate charge separation and migration while reducing the recombination of the electron–hole pairs in the TiO_2_ photocatalyst (Figure 2c and d and eq 1), thereby enhancing the efficiency of the photocatalyst and the degradation performance of the system (Figure 2e).
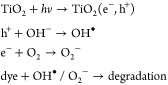
1

The PEC process was maintained by restricting current flow and using polydimethylsiloxane (PDMS)-coated Pt electrodes to prevent any electrocatalytic activity.

The Pt electrodes were attached to the TENG and submerged in the reactor containing the dye and TiO_2_ NPs. The TENG facilitated the degradation of 76% of the dye in 120 min, compared to only 26% in the same period without the TENG. Additionally, the degradation process accelerated with the increased frequency of impact force on the TENG, which generated a higher effective voltage per unit of time (Figure 2f).

**Figure 3:**
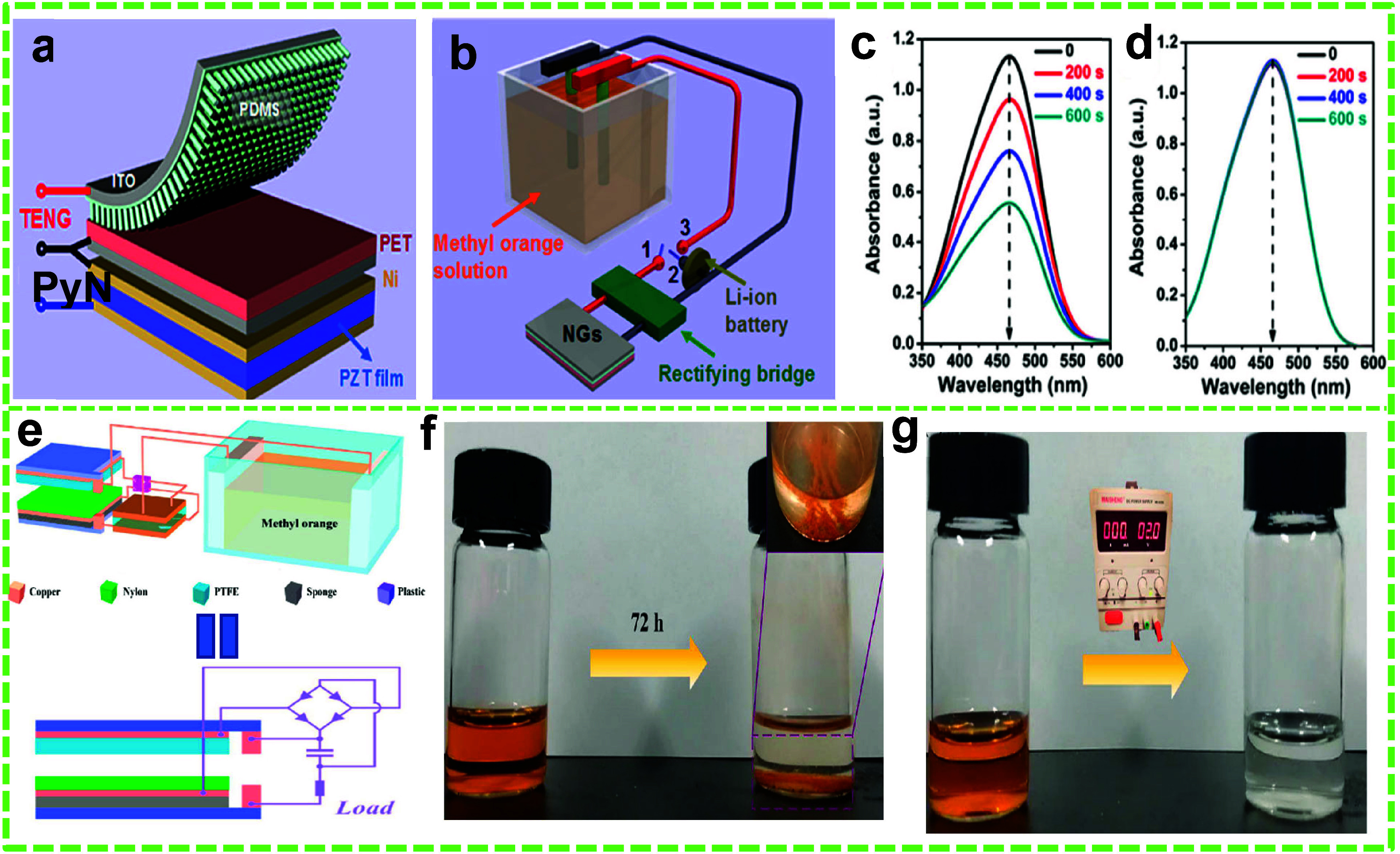
TENG-assisted EC degradation of MO dye. (a) Schematic of the CS-TENG and P_*y*_NG-based stacked-HNG. (b) Schematic of the EC degradation system containing MO dye. MO solution’s UV–vis absorption spectra (c) with and (d) without a 2 V Li-ion battery charged by the TENG. Reprinted with permission from ref (2). Copyright 2013 American Chemical Society. (e) Schematic of the PCVS-TENG with the EC system for MO degradation, (f) comparison of the MO solution at 0 h and after 72 h of degradation (the inset shows intermediate products sedimentation), and (g) degradation of the MO solution under 2 V constant voltage. Reprinted with permission from ref (141). Copyright 2020 Elsevier Ltd.

Electrocatalytic (EC) degradation relies on external power sources to break down chemical compounds. The EC degradation process is initiated by the generation of •OH radicals at the anode when an external power is applied. In wastewater treatment, the EC degradation of dyes like MO and rhodamine B has been extensively studied. However, the development of the technique is at a standstill due to the requirements of an external power source. To address this, self-powered EC degradation systems can be developed using TENGs to degrade organic pollutants in wastewater. Yang et al. demonstrated EC degradation of MO with the help of a hybrid nanogenerator (HNG) comprised of a CS-TENG and a P_*y*_NG.^2^ A stacked configuration was developed by placing the CS-TENG at the top and the P_*y*_NG at the bottom. (Figure 3a and b). The TENG’s friction layers comprised a PDMS nanoarray and a PET film, while the electrodes were made of indium tin oxide (ITO). Lead zirconate titanate (PZT) served as the pyroelectric element in the PyNG and was sandwiched between two nickel (Ni) electrodes.

The HNG can generate electricity from either the TENG or the P_*y*_NG individually or from both simultaneously. The EC degradation can be carried out in one of two ways: either by storing energy in a battery or by directly supplying electrical energy into the reaction bath to break down MO. In this system, a Pt electrode served as the anode, and sodium chloride (NaCl) was used as the electrolyte to enhance the conductivity of the MO solution. The degradation process is illustrated in eq 2
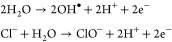
2where the electrocatalysis of the Pt anode results in the formation of hypochlorite ions (ClO^–^) and •OH radicals, which trigger oxidation and the degradation of the organic pollutants. The degradation process was conducted by charging a 2 V Li-ion battery with the TENG and connecting it to the Pt electrodes submerged in the reaction bath. Figure 3c shows the UV–vis absorption spectra of the MO over time. The absorption spectra remained unchanged over the same interval in a subsequent control experiment conducted without the TENG or the battery (Figure 3d). An experiment using the energy from both units was not performed. However, it was noted for the PyNG that the device can degrade 80% of the dye in 144 h when connected directly to the SPECs. Despite the potential of SPEC degradation, the proposed HNG requires a longer time to completely degrade the pollutants.

The efficiency of TENGs is often limited by their high matching impedance, low current output, and uncontrolled high voltage regardless of the load resistance. To address these issues, Xia et al. developed a pulse-controllable voltage source (PCVS)-based TENG (PCVS-TENG). This design includes a rectifier, capacitor, and unidirectional switch in the circuit system, allowing for efficient degradation of organic dyes.^141^ The electrodes in the TENG were made of Cu foils, while the frictional layers were comprised of PTFE and nylon films. The circuitry and working mechanism of the TENG are shown in Figure 3e. The EC degradation system used was similar to that employed by Yang et al. for MO,^2^ with a comparable degradation mechanism as depicted in eq 2. However, the results showed that using higher or selective voltages to degrade the MO solution led to the formation of intermediate products with low solubility, resulting in observable sediments (inset of Figure 3f) as degradation progressed in contrast to the degradation observed with a 2 V battery source (Figure 3g).

**Figure 4:**
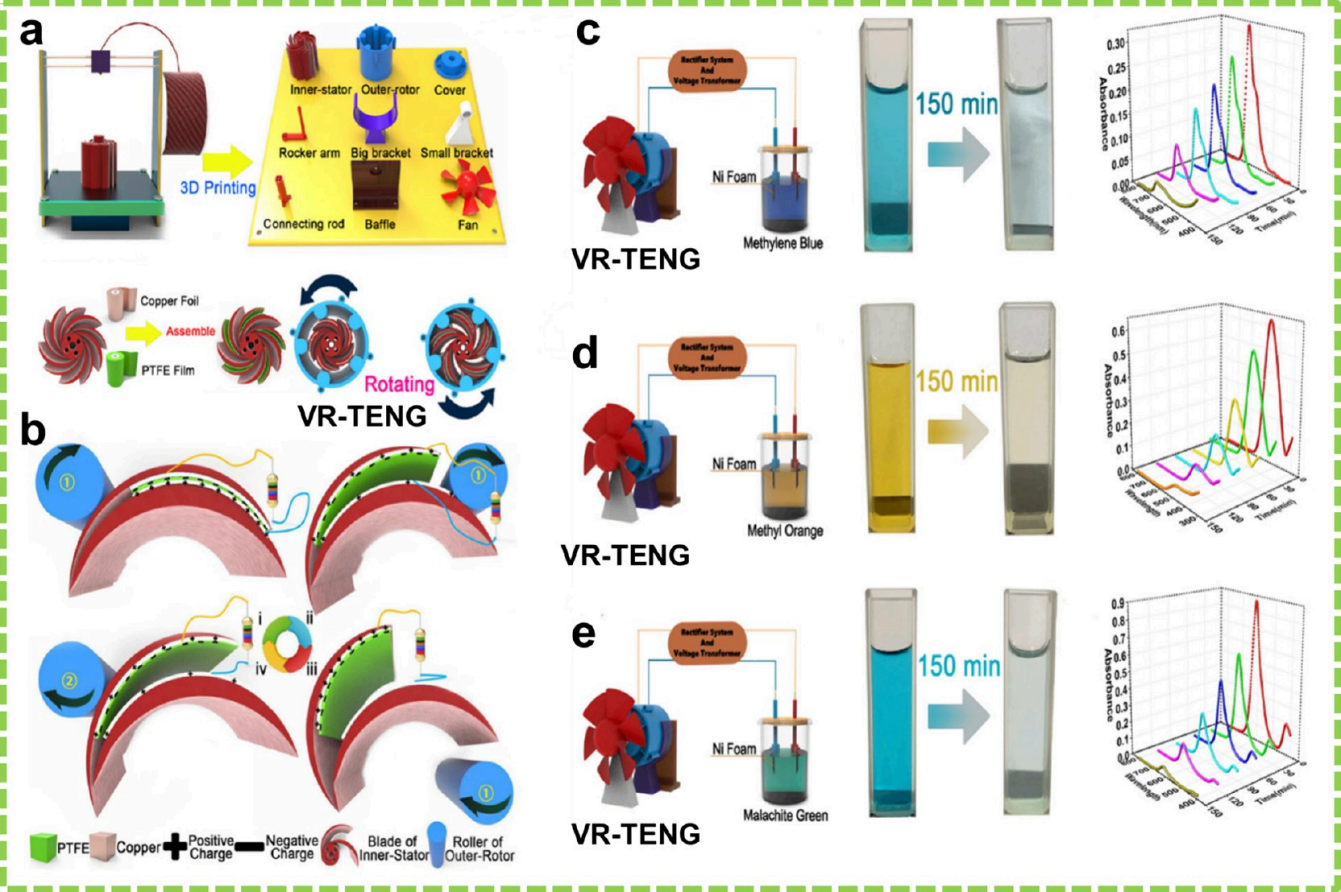
Fabrication and degradation performance of the CS mode-based VR-TENG. (a) Schematic of the fabrication process and (b) schematic of the working mechanism of the VR-TENG. Degradation of (c) methylene blue (MB), (d) MO, and (e) malachite green (MG) dyes for 150 min, along with their corresponding UV–vis absorption spectra. Reprinted with permission from ref (142). Copyright 2022 American Chemical Society.

Compared with the CS mode, rotational TENGs (R-TENGs) based on the FS mode have a lower peak current and a higher rate of frictional wear, and it is challenging to incorporate the CS mode into R-TENGs. Liu et al. developed a vortex-like R-TENG (VR-TENG) with 3D printing. This device features a hard rotor as the shell and an inner stator with a hierarchical structure of alternating 3 and 5 mm flexible blades.^142^ Thermoplastic elastomer (TPE) and poly(lactic acid) (PLA) filaments, respectively, were used to print the flexible stator and the hard rotor (Figure 4a). The inner sides of the short blades had Cu foil back electrodes with PTFE as the frictional layers, while the top sides of the long blades featured Cu foil as both the frictional layers and electrodes. The hard rotor contained rollers, which facilitated contact separation between the frictional layers during rotation (Figure 4b).

The VR-TENG’s efficacy was demonstrated by the degradation of MO, MB, and MG using Ni and Cu foams as the anode and cathode, respectively. The system contained 0.07 M Na_2_SO_4_ as the electrolyte and 10 mg L^–1^ dye solutions of MO, MB, and MG. The degradation experiments were conducted for 150 min using a rectified and transformed electrical output. The removal efficiencies for MO, MB, and MG were found to be 88.9%, 91.7%, and 94.1%, respectively (Figure 4c–e).

##### 4.1.1.2 Rhodamine B (RhB)

**Figure 5:**
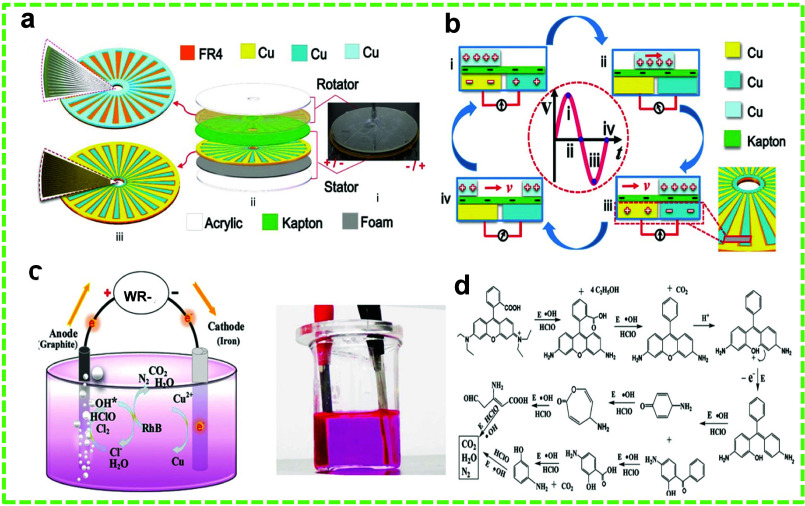
WR-TENG-assisted EC degradation of RhB dye. (a) Schematic and (b) working mechanism of the WR-TENG for RhB removal. (c) Schematic of the EC system for RhB degradation and (d) the proposed pathway for stepwise RhB degradation. Reprinted with permission from ref (40). Copyright 2016 John Wiley & Sons.

Chen et al. developed a wastewater wave-harnessing R-TENG (WR-TENG), which showed great promise in removing almost 100% RhB pollutant in 15 min from an initial concentration of 100 ppm.^40^ The schematics of the TENG and working mechanism are presented in Figure 5a and b, respectively.

The electrochemical unit for RhB treatment comprised a graphite anode, an iron cathode, and 20% (w/v) NaCl as the electrolyte to enhance ionic strength. The degradation mechanism is based on electrolysis: H_2_O and Cl^–^ are transformed into •OH and ClO^–^ at the anode. These oxidative species then degrade RhB into CO_2_, H_2_O, N_2_, and other byproducts (Figure 5c). However, if the initial RhB concentration is high, intermediate organic products, such as carboxylic acids, may also be present. RhB undergoes degradation through a series of parallel and subsequent reactions, as illustrated in Figure 5d. This process is essential for effectively removing this pollutant from the environment. In this context, WR-TENG provided the necessary power for electrochemical (EC) degradation, which was obtained through successive transformation and rectification. This mechanism underscores the potential of WR-TENGs to power sustainable and efficient pollutant degradation processes.

In another study, Feng et al. developed a SPEC that integrates the EF process with a wind-driven R-TENG (WDR-TENG) to degrade RhB dye.^143^ In the EF process, •OH radicals are the reactive species responsible for degrading organic pollutants. A major drawback of EF is its requirement for additional oxygen, energy sources, and acidic conditions, which necessitates further treatment. In this regard, Feng et al. successfully demonstrated a SPEC capable of degrading RhB and MB in a neutral environment without the requirement of an external power source. This highlights the potential of SPECs to provide sustainable and energy-efficient solutions for pollutant degradation, marking significant advancements in environmental remediation. In this study, the electrolyte was a 0.1 M aqueous solution of NaCl with 0.2 mM FeSO_4_. The carbon black (GF3) cathode was made as the electrocatalyst, with PTFE-modified graphite, felt as the cocatalytic component, and binder, while a Pt sheet was used as the anode.

**Figure 6:**
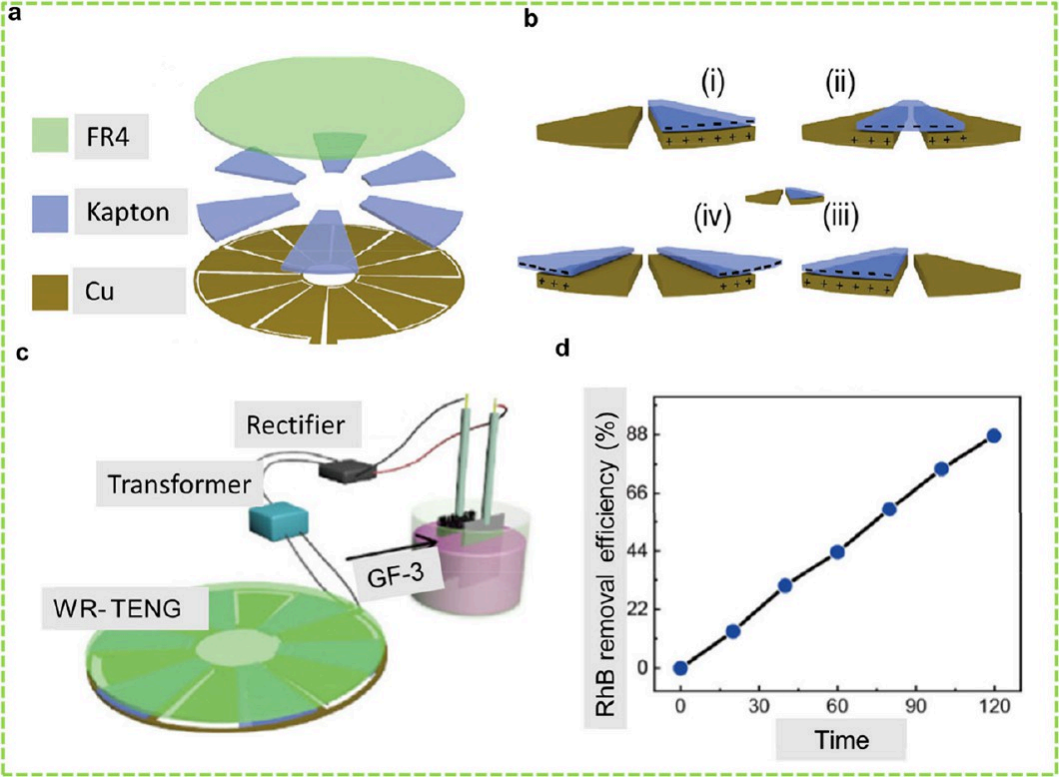
WDR-TENG-assisted EC degradation of RhB dye. (a) Schematic and (b) the working mechanism of the WDR-TENG for RhB removal. (c) Schematic of the EC system for RhB degradation and (d) the removal efficiency of RhB by the WR-TENG as a function of time. Reprinted with permission from ref (143). Copyright 2019 Springer Nature.

The R-TENG’s stator, which also functioned as one of the frictional layers, was composed of Cu segments arranged in a radial array. The rotor was made from an FR4 disc substrate with annular sectors of Kapton film serving as the other frictional layer (Figure 6a–c). The degradation mechanism involves the generation of H_2_O_2_, •OH, and chlorine species. At a concentration of 3 mg L^–1^ and a volume of 25 mL, the R-TENG removed 87.5% of RhB in 120 min with the wind speed set at 6.2 m s^–1^ (Figure 6d).

**Figure 7:**
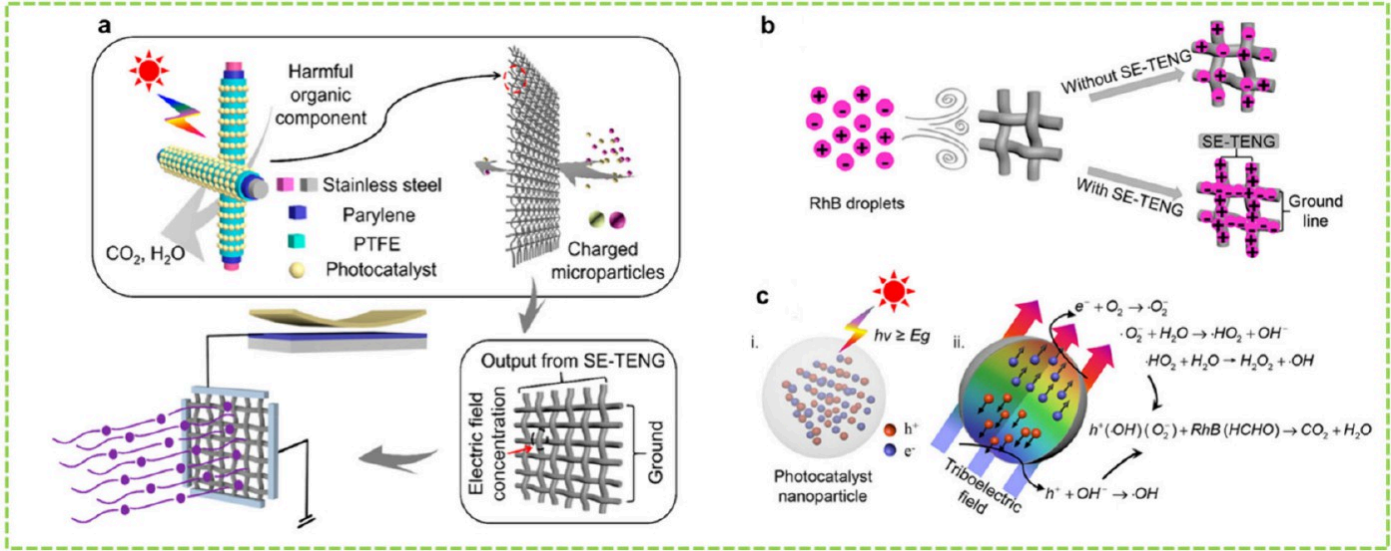
SE-TENG-assisted adsorption and PEC degradation of RhB dye. (a, b) Schematics of the SE-TENG-powered electrostatic absorption of RhB or HCHO. (c) The PEC degradation mechanism of RhB and formaldehyde powered by the SE-TENG. Reprinted with permission from ref (13). Copyright 2017 American Chemical Society.

Feng et al. utilized electrostatic adsorption and TENG-enhanced photocatalysis techniques to develop a self-powered pollutant filter mesh.^13^ The filter mesh was supported on an acrylic frame and was constructed of polymer-coated stainless steel wires. The metal wires were first coated with Parylene using vacuum vapor deposition and subsequently with PTFE using dip coating techniques. The PTFE layer was then dip-coated in either P25 or a Pt-added P25/PTFE suspension to add photocatalytic capability. The vertical and horizontal wires in the filter mesh (Figure 7a) were connected to two different Al strips and were spaced apart by dielectric layers. The filter mesh was connected between an SE-TENG and its ground electrode, as shown in Figure 7a, through the Al strips. The SE-TENG charged the filter network in response to mechanical stimulation, generating a strong electric field across the mesh. This field enabled the adsorption of pollutants, including polar organic pollutants and particulates, onto the filter wires. To demonstrate the TENG-enhanced electrostatic adsorption effect, RhB was sprayed onto the filtering network with and without SE-TENG activation. The dye particles acquired charges from friction with compressed air during spraying. When the SE-TENG was not activated, most dye particles passed through the filter, with only a few depositing randomly (Figure 7b).

With the SE-TENG, a strong electric field was generated, and the Coulomb force caused the dye particles to be absorbed into the filter. When connected to the SE-TENG, the filter could adsorb the same amount of RhB in one min compared to 15 min without the SE-TENG. Additionally, the degradation time of RhB was reduced by 50% with the SE-TENG. Furthermore, the degradation of formaldehyde was doubled due to the enhanced absorption of polar formaldehyde molecules. The degradation mechanism is illustrated in Figure 7c.

Recent studies have focused on using ferroelectric materials to boost the current density of the TENGs. Due to their excellent dielectric properties from spontaneous polarization, ferroelectric materials are ideal for enhancing TENG performance. Wang et al. developed a TENG in CS mode using ferroelectric La_0.05_Fe_0.05_ codoped ZnO nanoarrays as the positive layer and PDMS as the negative layer, with Zn and Cu serving as the corresponding electrodes.^88^ The positive frictional layer’s superior dielectric and ferroelectric properties significantly enhanced the current density. The SPEC unit contained RhB dye, Cu foils as the electrodes, and NaCl as the electrolyte. Although the volume of the solution and the dye concentrations were not disclosed, the unit achieved 100% RhB removal efficiency in 5 h.

##### 4.1.1.3 Methyl Red (MR)

**Figure 8:**
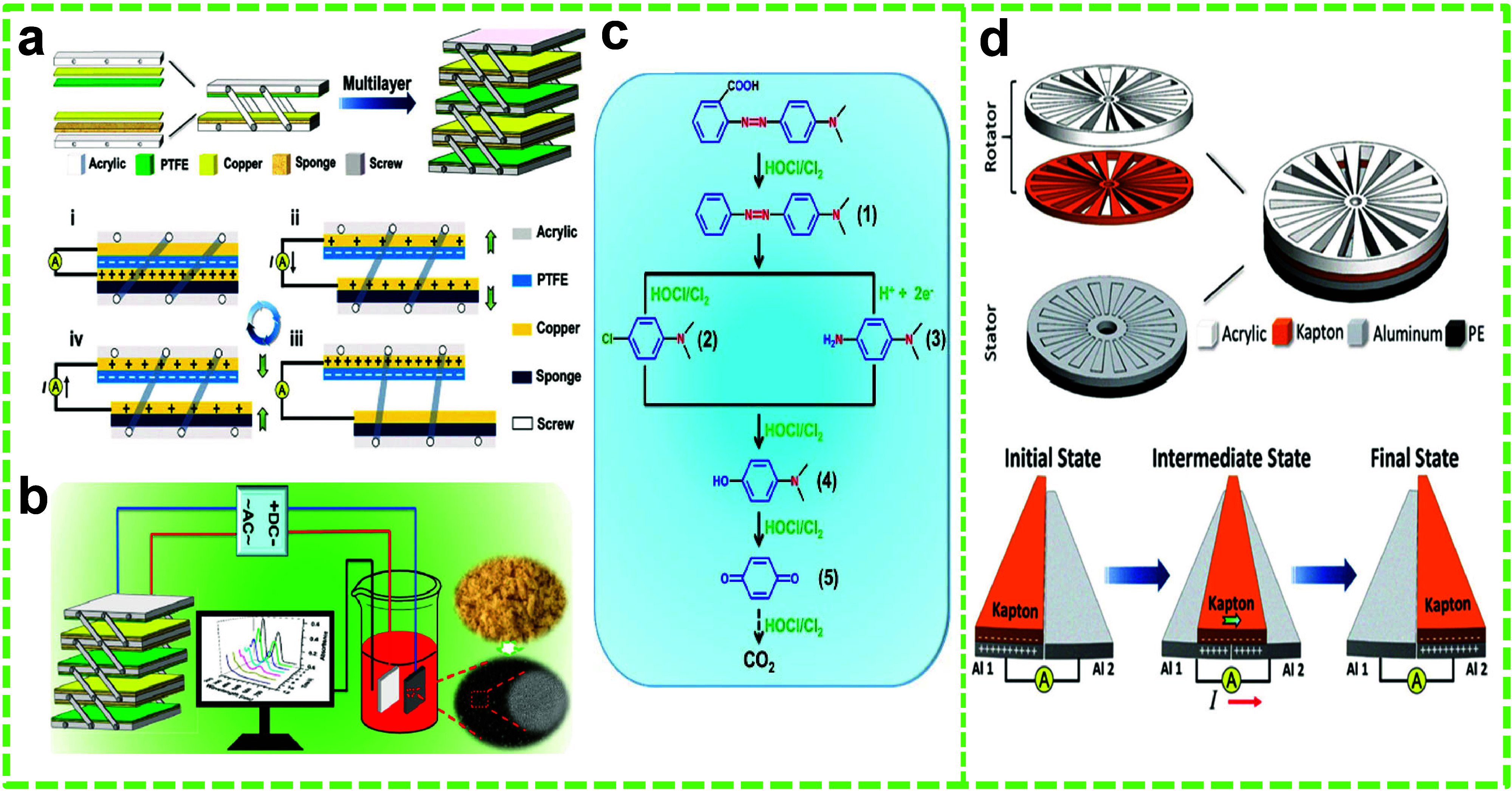
TENG-assisted EC degradation of MR and 4-aminoazobenzene dyes. (a) Schematic and the working mechanism of the ML-TENG for MR removal. (b) Schematic of the EC system for MR degradation and (c) the proposed degradation mechanism of the MR dye driven by the ML-TENG. Reprinted with permission from ref (83). Copyright 2017 American Chemical Society. (d) Schematic and the working mechanism of the R-TENG for 4-aminoazobenzene removal. Reprinted with permission from ref (10). Copyright 2017 American Chemical Society.

The EC technique typically uses electrodes made from materials like Pt, Ti/PbO_2_, Cu mesh, and Fe. While Pt-based electrodes are effective catalysts, they are expensive and have a limited supply. Recent research has highlighted the effectiveness of carbon-based electrodes in treating organic pollutants. Gao et al. developed a carbon-based electrode derived from bean curd and paired it with a multilayer linkage TENG (ML-TENG) operating in the CS mode. This setup was effectively used to degrade MR pollutants from wastewater, demonstrating the potential of using sustainable and readily available materials in environmental remediation efforts. It also underscores the versatility of TENGs in various operational modes for pollutant degradation.^88^ The ML-TENG, resembling a laboratory jack, was assembled in steps with alternating frictional layers, as shown in Figure 8a. In the ML-TENG, PTFE and Cu films served as the primary frictional layers. A sponge layer was included to ensure intimate contact between the frictional layers, and PTFE was precharged to increase the charge density for better electrical output. During operation at 600 rpm, the EC process was conducted using a 0.5 mol L^–1^ HCl electrolyte and a 10 mg L^–1^ MR concentration (Figure 8b). The production of HOCl/Cl_2_ at the anode and H__2__/H_2_O at the cathode initiates the MR degradation mechanism, which continues until mineralization to CO_2_ (Figure 8c). The device achieved nearly 100% degradation in 160 min.
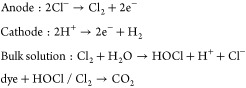
3

In a recent study, Gao and his team used an R-TENG to degrade 4-aminoazobenzene.^10^ The rotor of the R-TENG was constructed from radially arrayed sectors of Kapton film on an acrylic substrate, while the stator consisted of an Al composite panel with two complementary patterned electrode networks on the same plane separated by fine gaps. The schematic and working mechanism of the R-TENG are described in Figure 8d. The anodic and cathodic reactions are similar to those depicted in eq 3 for MR degradation.^83^ They also demonstrated that the dye can be converted to oligomers such as permigraniline, emeraldine, and leucoemeraldine by controlling the oxidation potential. The process was extended to 2-(4-dimethylaminophenylazo) benzoic acid, achieving 98.4% degradation within 600 min.

##### 4.1.1.4 Methyl Yellow (MY)

**Figure 9:**
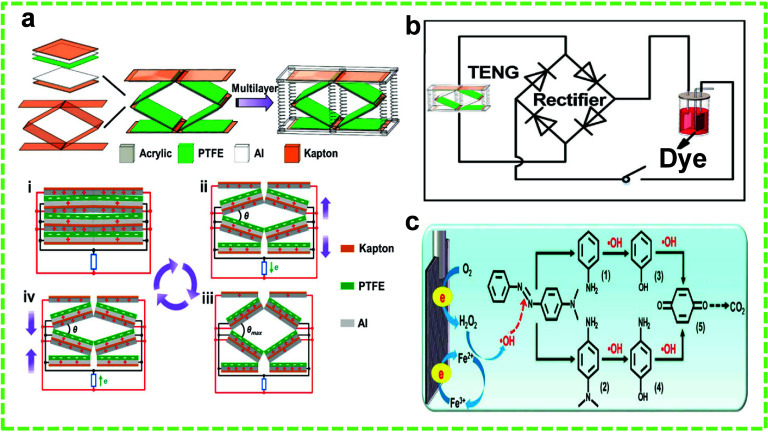
TENG-assisted EC degradation of MY and 4-dimethylaminoazobenzene dyes. (a) Schematic and the working mechanism of the rhombic ML-TENG for MY removal, (b) schematic of the EC system for MY degradation by the rhombic ML-TENG, and (c) the proposed mechanism of 4-dimethylaminoazobenzene degradation by the ML-TENG driven EF system. Reprinted with permission from ref (132). Copyright 2018 Elsevier Ltd.

A similar carbon-based electrode in combination with the EF method was used by Gao et al. to demonstrate the degradation of MY using an ML-TENG in CS mode.^132^ In this setup, the friction layers were made of PTFE and aluminum (Al) films, with PTFE being charged and injected to enhance output. The TENG was constructed with six friction layers arranged in a rhombic grid-like structure. The charge injection increased the voltage and current by 2.5× and 3×, respectively. The schematic and working mechanism of the TENG are shown in Figure 9a.

The carbon-based electrode materials, produced from long beans and featuring meso/macroporous structures, offer effective channels for the mass transfer of O_2_ to the cathode, enhancing its reduction to H_2_O_2_. The electrochemical unit consisted of Pt foil as the anode and a stainless-steel mesh covered with carbon materials as the cathode, with sulfuric acid used for pH control and Na_2_SO_4_ as the electrolyte. In 120 min, the device achieved 99% degradation of the MY dye. Additionally, it degraded Basic Orange 2 dye by 98% in 90 min, demonstrating its versatility in degrading different pollutants. The application of the EF technique reduced the degradation time to 70 min, compared to 110 min with conventional electrochemical methods. The device effectively degraded the dyes through in situ production of H_2_O_2_ via O_2_ reduction and subsequent production of •OH radicals. The degradation process of the dye is illustrated in Figure 9b and c.

##### 4.1.1.5 Methylene Blue (MB)

**Figure 10:**
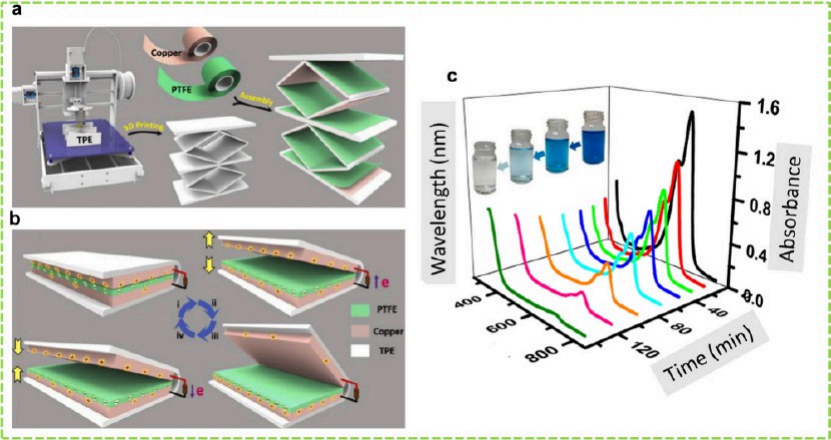
ML-TENG-assisted EC degradation of MB dye. (a) Schematic and (b) the working mechanism of the rhombic ML-TENG for MB removal. (c) UV–vis spectra of MB degradation driven by the rhombic ML-TENG. Reprinted with permission from ref (131). Copyright 2020 Elsevier Ltd.

3D printing is a promising additive manufacturing technique for creating soft and pliable structures with complex architectures. This advanced technique can also be utilized to fabricate TENGs with greater design flexibility.^144^ Tian et al. introduced a rhombic ML-TENG in CS mode using 3D printing and integrated it with the EF system to degrade MB dye.^131^ In this case, porous carbon materials from biomass were used as the cathode catalysts. The substrate for the TENG was printed with a TPE filament. As shown in Figure 10a and b, the substrate included two rhombic units that were held up by the top, bottom, and middle 3D plates. Herein, Cu foils were used as the positive friction layers and electrodes, while PTFE films served as the negative frictional layers. Furthermore, charges were injected into the PTFE layers to improve the TENG’s performance efficiency. With the TENG providing the electricity, the system achieved a 97% removal efficiency of MB in 140 min (Figure 10c).

**Figure 11:**
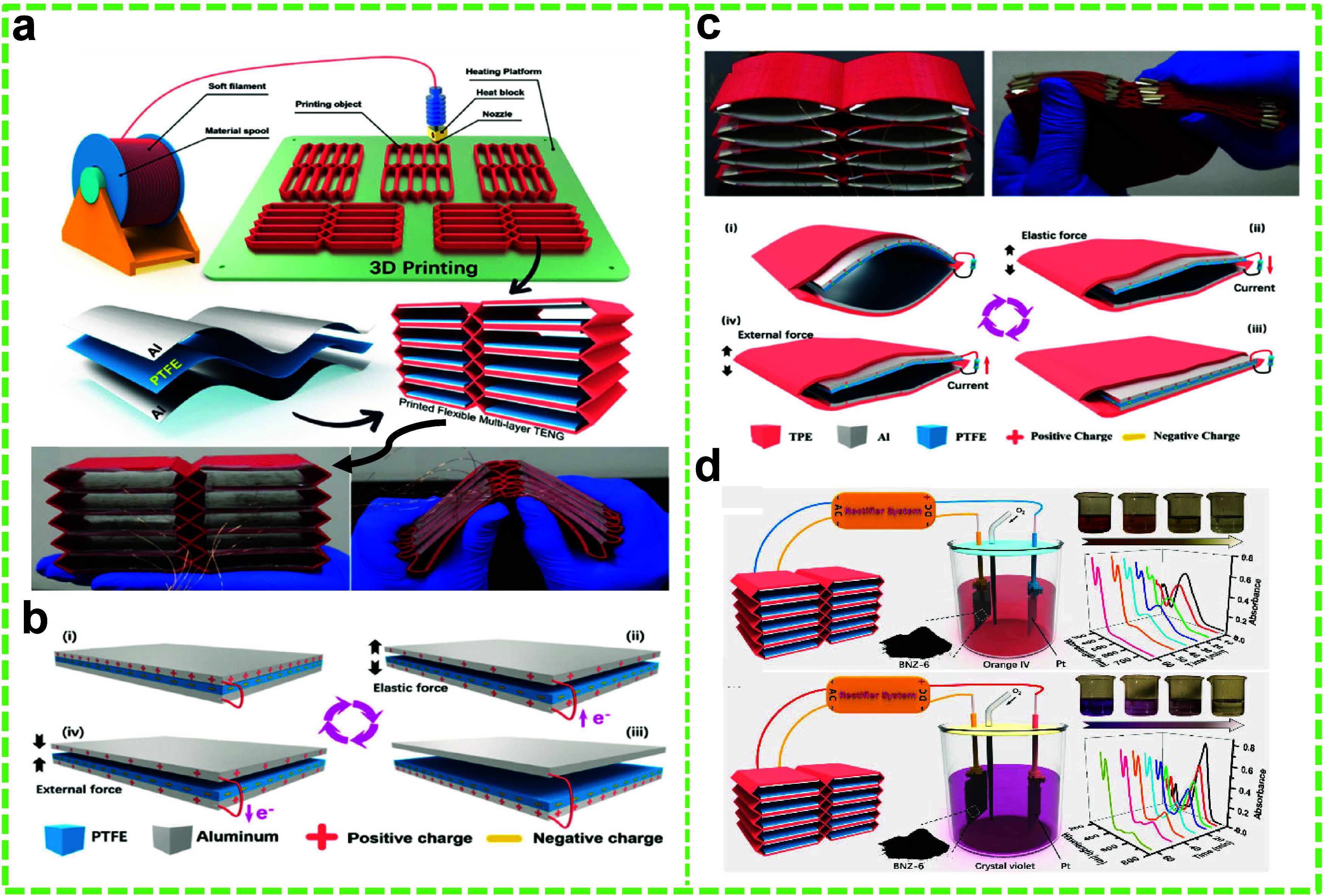
ML-TENG-assisted EC degradation of MB, orange IV, and crystal violet dyes. (a)Schematic and (b) the working mechanism of the 3D printed ML-TENG for MB removal. Reprinted with permission from ref (130). Copyright 2021 Elsevier Ltd. (c) Schematic and (d) the working mechanism of the 3D printed ML-TENG for orange IV and crystal violet removal. Reprinted with permission from ref (129). Copyright 2021 Elsevier Ltd.

In a recent study, Zhu et al. degraded MB dye using an EF system with a highly efficient oxygen-containing functional-group-rich N-doped hierarchical porous carbon material as the cathode catalyst. These structural features endow the cathode with higher adsorption and degradation capabilities. The TENG was similarly constructed by using 3D printing technology, employing TPE to design a flexible construct with eight CS-TENG units. The EF system comprised Na_2_SO_4_ (0.05 mol L^–1^, pH = 2.0) and FeSO_4_ (0.2 mmol L^–1^), with a catalyst-coated glassy carbon working electrode and a Pt sheet counter electrode. Each unit featured an Al foil electrode with a triboelectric layer attached to the lower surface of the 3D-printed construct, while PTFE served as the other triboelectric layer with back Al foil-based electrodes anchored to the top surface of the unit (Figures 11a and b).^129^

The study found a degradation efficiency of 98.1% in 58 min; however, the concentration of MB in the solution is not mentioned. It was observed that the presence of oxygen-containing functional groups positively correlated to the degradation performance. Additionally, the researchers extended the application of the 3D TENG to a construct with 10 units using the same cathode catalysts to degrade Orange IV and Crystal Violet. In this setup, Na_2_SO_4_ and FeSO_4_ were used, with a catalyst-containing stainless steel mesh as the working electrode and a Pt sheet as the counter electrode (Figure 11c).^130^ The degradation efficiencies were 96.0% for Crystal Violet (10 mg L^–1^) and 95.4% for Orange IV (20 mg L^–1^) within 60 min (Figure 11d).

##### 4.1.1.6 Miscellaneous Dyes

Shen and Fu et al. demonstrated the PEC degradation of Brilliant Green (BG) and Direct Blue (DB) using an R-TENG. The employed photocatalyst was based on carbon-dotted TiO_2_ sheets doped with 3D graphene oxide (3DGA@CDs-TNs. In the typical setup, the R-TENG’s rotor featured radially arranged Cu segments as the frictional layer, while the stator used PTFE as the other frictional layer, with conductive electrode segments arranged radially (Figure 11).^145^ The TENG-driven PEC system achieved 88.26% degradation of BG in 40 min and 89.6% degradation of DB in 1.5 h. In comparison, the degradation efficiencies without the TENG were 81.66% for BG in 2 h and 73.23% for DB in 3 h. The enhanced efficiency of the TENG/3DGA@CDs-TNs system is attributed to the applied electric field, which prevents the recombination of electron–hole pairs and is further improved by the electron collection and transfer capabilities of 3DGA. The PEC experiments used 5 ppm BG and 20 ppm DB, with a graphite anode, iron cathode, and 10% NaCl electrolyte powered by rectified electric power.

#### 4.1.2 Gummy Materials

Natural biopolymers found in plant cells, such as lignin, hemicellulose, and pectin, have adhesive-like properties. While they are not pollutants themselves, their regeneration and degradation can produce harmful compounds. When discharged as byproducts from industries (such as paper production, biofuel, and biomass processing) or in agricultural burn residues, they can become pollutants, impacting aquatic ecosystems and air quality. Incineration and combustion of these biopolymers can also pose health risks. Managing and treating these biopolymers sustainably is crucial.^146^

**Figure 12:**
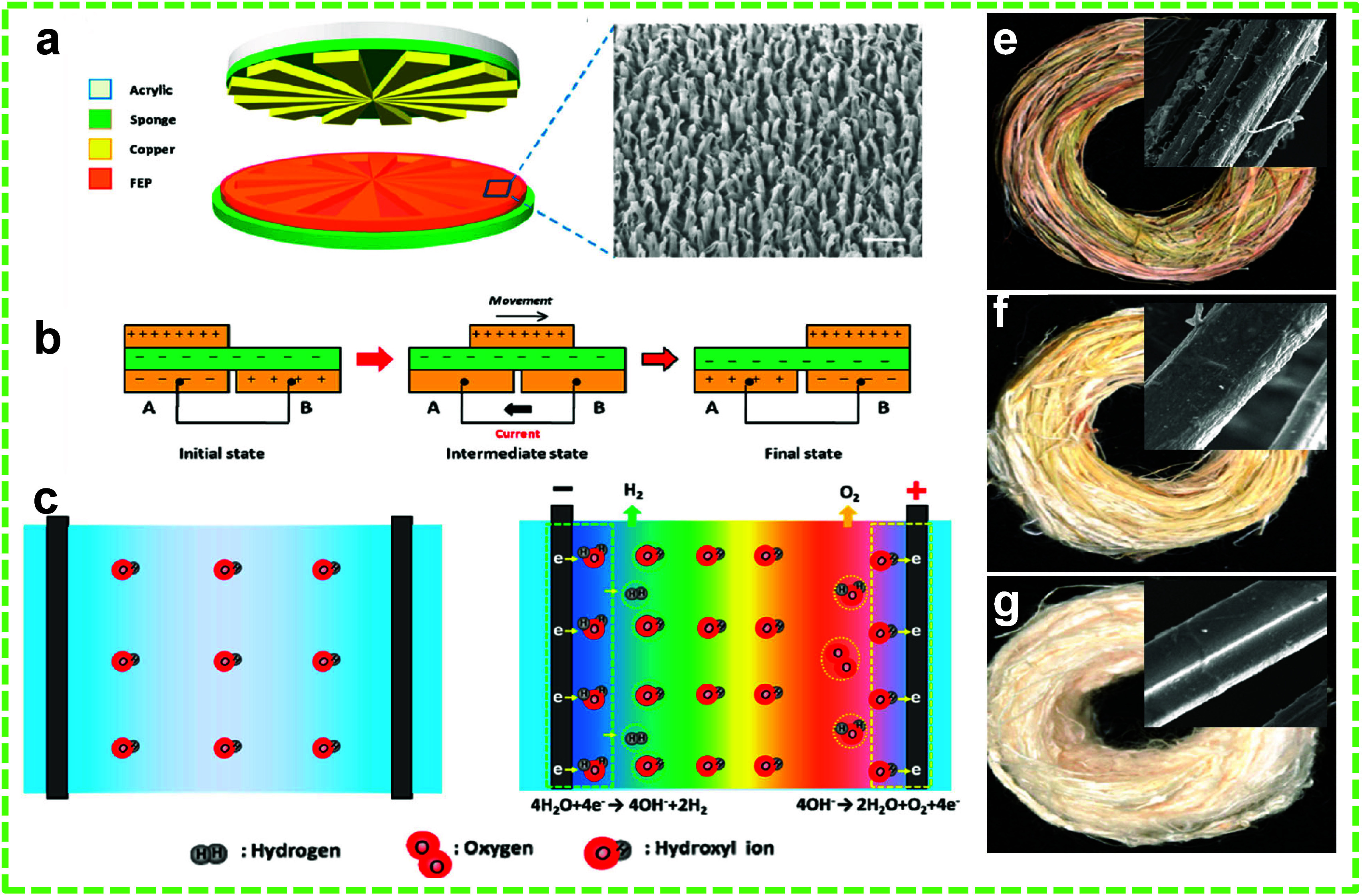
TENG-assisted removal of gummy materials from natural fibers. (a) Schematic (with inset SEM image of FEP nanowires) and (b) the working mechanism of the WR-TENG for the degumming of the ramie fiber. The mechanism of degumming the ramie fiber (c) without the WR-TENG and (d) with the WR-TENG. Ramie fibers (e) without treatment, (f) treated without WR-TENG, and (g) treated with WR-TENG (with inset SEM images). Reprinted with permission from ref (82). Copyright 2016 Elsevier Ltd.

Li et al. demonstrated the removal process of gummy materials, such as hemicellulose, lignin, and pectin, from the extraction of natural fibers (i.e., ramie) through electrolysis powered by a WR-TENG.^82^ The current degumming procedure is costly, time-consuming, and energy-intensive. It also has a high operating cost, suffers from pollution, and produces wastewater. The innovation in this study aimed to accelerate the degumming rate while minimizing chemical usage, resulting in fibers with enhanced quality compared to those obtained via conventional methods. The WR-TENG was constructed using Cu and nanopatterned FEP as frictional layers in their setup. The stator disc was laser-cut to create small trenches with complementary patterns, followed by the deposition of a Cu layer and the connection of lead wires to the complementary Cu patterns for power collection. A thin FEP layer was applied to the Cu electrodes, while the rotor was coated with Cu to act as the friction layer against the FEP. The rotor was connected to a water turbine for degumming and degradation studies. The schematic and the working mechanism of the WR-TENG are shown in Figure 12a and b. Traditionally, alkali is used in degumming to generate OH^–^ ions, which break down gummy polysaccharides into soluble units. With the new technique, an electrochemical unit comprising a Ti/PbO_2_ anode, a Ti cathode, and a degumming solution was self-powered by the WR-TENG to accelerate the degradation of gummy materials (Figure 12c-d). Under the electric field, cations move toward the cathode, while the anion moves toward the anode, resulting in a large number of OH^–^ in the degumming solution. The resultant fiber exhibited an improved crystallinity index (from 68.2% to 86.96%), tenacity (4.34 cN/dtex), elongation (from 1.89% to 3.25%), and fineness (from 1352 to 1851 N m) compared to fibers processed using traditional methods. Additionally, •OH generated at the anode would electrocatalytically oxidize the gummy materials. Under a current and voltage of 3.5 mA and 10 V, respectively, the system could clean 90% of the pollutants in the wastewater. The surfaces of untreated and treated ramie fibers are shown in Figures 12e–g.

#### 4.1.3 Antibiotics

Antibiotic pollution is a pressing global issue. Overuse and residual discharge of antibiotics harm human health and the environment. Pharmaceuticals, expired medicine, and hospital waste contribute to antibiotic pollutants in water bodies, posing challenges to environmental health.^147−153^

**Figure 13:**
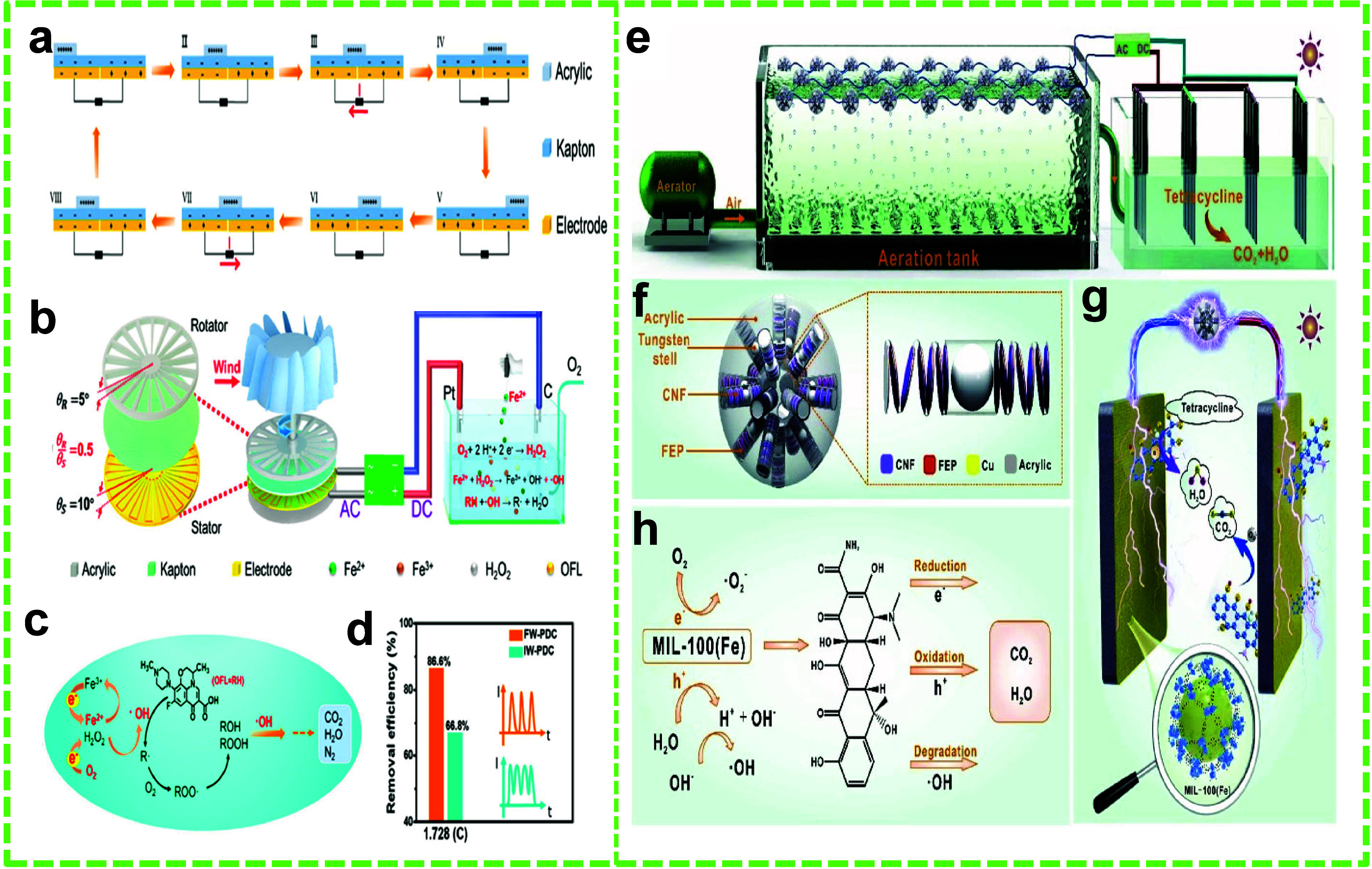
TENG-assisted degradation of antibiotics. (a) Working mechanism and (b) schematic of the R-TENG for levofloxacin degradation. (c) Degradation of the levofloxacin in the EF system facilitated by the R-TENG. (d) Removal efficiency of FW-PDC vs IW-PDC. Reprinted with permission from ref (154). Copyright 2021 American Chemical Society. (e) Schematic of the experimental setup for the degradation of tetracycline. (f) Schematic of the C-TENG’s construction. (g, h) The degradation mechanism of tetracycline. Reprinted with permission from ref (155). Copyright 2021 Elsevier Ltd.

Levofloxacin, a fluoroquinolone antibiotic, is widely used to treat bacterial and respiratory infections. However, it is not completely metabolized in the body and escapes into the environment through urine and feces. This mechanism can lead to chronic toxicity and antibiotic-resistant bacteria, which are detrimental to humans and the environment. To address this, Liu and his team designed an R-TENG to power the EF process for removing levofloxacin.^154^ However, the efficiency of such electrochemical (EC) systems can be compromised by electrode passivation. Studies indicate that pulsed direct current (PDC) can mitigate electrode passivation and enhance the system’s efficiency. Despite its potential, the full-wave PDC (FW-PDC) modified R-TENG is rarely used due to phase superposition issues caused by multiple parallel electrodes operating simultaneously within the device. These challenges can be addressed by carefully optimizing the angle ratio between each rotor and stator in R-TENGs. The R-TENG’s design, construction, and working mechanism are depicted in Figure 13a and b. The rotor is made of Kapton film with complementary electrodes on the back, while the stator consists of 18 radially arranged acrylic segments. The triboelectrification between Kapton and acrylic generates the energy required for the EF process. The electrolysis system employs Pt as the anode and carbon as the cathode, with 0.05 M Na_2_SO_4_ as the electrolyte. Introducing O_2_ and Fe^2+^ leads to the generation of •OH radicals through redox reactions, which effectively degrade the organic pollutant into H_2_O, CO_2_, and N_2_ (Figure 13b and c). Compared to the incomplete wave pulsed direct current (IW-PDC) R-TENG, the FW-PDC R-TENG demonstrates a 30% higher removal efficiency, highlighting its potential for more effective pollutant degradation (Figure 13d).

In addition, acidic conditions proved to be more effective than alkaline conditions in this system. This is because in alkaline conditions the presence of OH^–^ ions consumes a significant amount of •OH radicals, which in turn reduces the overall yield of these reactive species. Under acidic conditions, the degradation of levofloxacin was nearly complete, with a reduction from an initial concentration of 1 ppm to almost zero within 1.5 h when operating with a rectified current at a wind speed of 2.8 m s^–1^.

Mo et al. demonstrated the degradation of tetracycline using a corona-shaped triboelectric nanogenerator (C-TENG) in CS mode integrated with a metal–organic framework (MOF) based photocatalyst in a PEC system.^155^ The aeration process, commonly employed for tetracycline treatment, produces water waves that often go unused. In this study, Mo et al. harnessed the mechanical energy generated by these water waves by using the C-TENG to enhance the degradation process of tetracycline, breaking it down into water (H_2_O) and carbon dioxide (CO_2_). The PEC system included an aeration tank, 18 units of C-TENG, a rectifier, and a photoreactor (Figure 13e and f). The C-TENG was fabricated from nanofibrillar cellulose and FEP frictional films, and Cu foils served as electrodes. The process and mechanism of tetracycline degradation via the PEC system are illustrated in Figures 13g and h. In the experiment, 100 mL of a tetracycline solution with a concentration of 10 mg L^–1^ was treated using 50 mg of MOF photocatalyst, with the C-TENG supplying rectified current generated at a water wave acceleration of 5 m s^–2^. The removal efficiency of tetracycline reached 92.72% within 60 min, which was significantly higher than the 86.37% efficiency observed without using the C-TENG.

#### 4.1.4 Pesticides

Pesticides, encompassing insecticides, herbicides, and fungicides, are a diverse group of compounds used to combat pests. They play a crucial role in enhancing the growth of plants, crops, and agricultural products. However, the residual effects of these substances on resources such as surface water and groundwater, due to off-site movement, leaching, and runoff, have raised concerns. Pesticides based on organochlorine, organophosphate, carbamates, and pyrethroids and their residues can have severe impacts on humans and wildlife. Exposure through ingestion, inhalation, and skin contact can lead to conditions such as cancer, immune system deficiencies, congenital disabilities, and pulmonary hematological disorders.^156,157^

**Figure 14:**
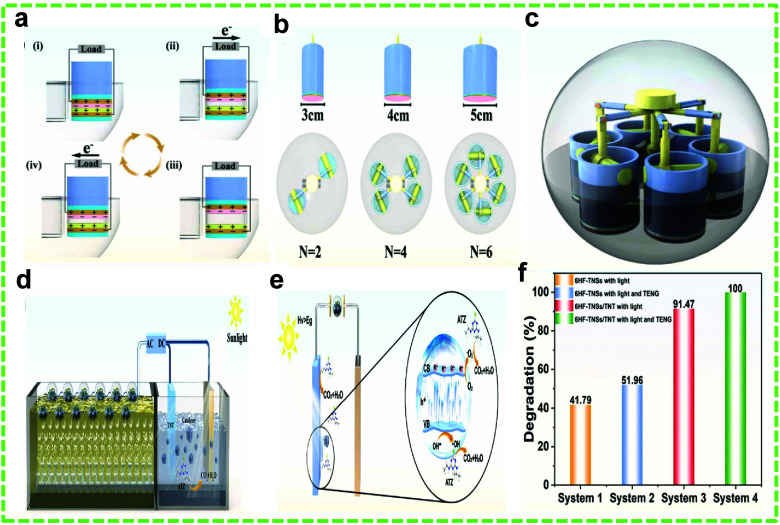
TENG-assisted degradation of pesticides. (a–c) Schematic of the CS-TENG for the removal of ATZ. (d) Schematic of the PEC system for the removal of ATZ. (e) PEC degradation mechanism of ATZ by the CS-TENG. (f) The degradation of ATZ under different experimental parameters. Reprinted with permission from ref (163). Copyright 2022 Elsevier Ltd.

Atrazine (ATZ), a common weed-controlling pesticide, has a half-life of 63 days in surface water. Previous research indicated that residual ATZ concentrations in American and Indian rivers ranged from 0.5 to 11.3 g L^–1^ and up to 22.0 mg L^–1^, respectively. It might also contain carcinogenic agents detrimental to human health.^158−162^ Dong et al. demonstrated an effective PEC system to degrade ATZ using a piston-like TENG constructed of six spherical CS-TENGs.^163^ Each TENG unit was designed so that contact separation between nylon and FEP films occurred when water waves struck individual TENG units. In this case, the electrodes and substrate for each TENG unit were Cu foils and PVC, respectively (Figure 14a–c). Photocatalysts, 6HF-TiO_2_ nanosheets (6HF-TNS), and loaded TiO_2_ nanotubes (TNT) were used as the photoanode. The •OH, h+, and O_2_^–^ radicals were produced on the photoelectrode surface of TNT and 6HF-TNSs due to electron and hole separation under illumination. The TENGs’ rectified PDC electric field made it harder for electrons to recombine with holes, encouraging the production of •OH and h+. Consequently, reactive species interacted with ATZ and degraded it into intermediate products or finally into CO_2_ and H_2_O (Figure 14d and e). The PEC experiments were carried out in a 150 mL photoreactor with 5 μmol L^–1^ ATZ for 30 min. Without the external field, the degradation rate in 30 min was 91.47% as opposed to 100% with the bias. Figure 14f shows the different 6HF-TNS systems and their respective degradation percentages.

#### 4.1.5 Urine ontents

Urban sewage is the primary source of pathogens, including bacteria, viruses, and fungi, which severely threaten the environment and human health. Urine is a massive source of nutrients (e.g., N, P, and K) for crops if appropriately recycled. Despite making up only 1% of domestic wastewater, it accounts for 79%, 47%, and 71% of the total N, P, and K, respectively, in the overall municipal wastewater.^164−166^ However, micropollutants in urine need to be decontaminated before being released into the environment. Furthermore, hospital urine contains 3-fold more microorganisms than hospital wastewater, which poses a significant health risk and energy waste in the following sewage treatment. The current wastewater treatment techniques produce mutagenic and carcinogenic organo-chlorinated species and have high energy costs and low efficiency.^87,167−169^

**Figure 15:**
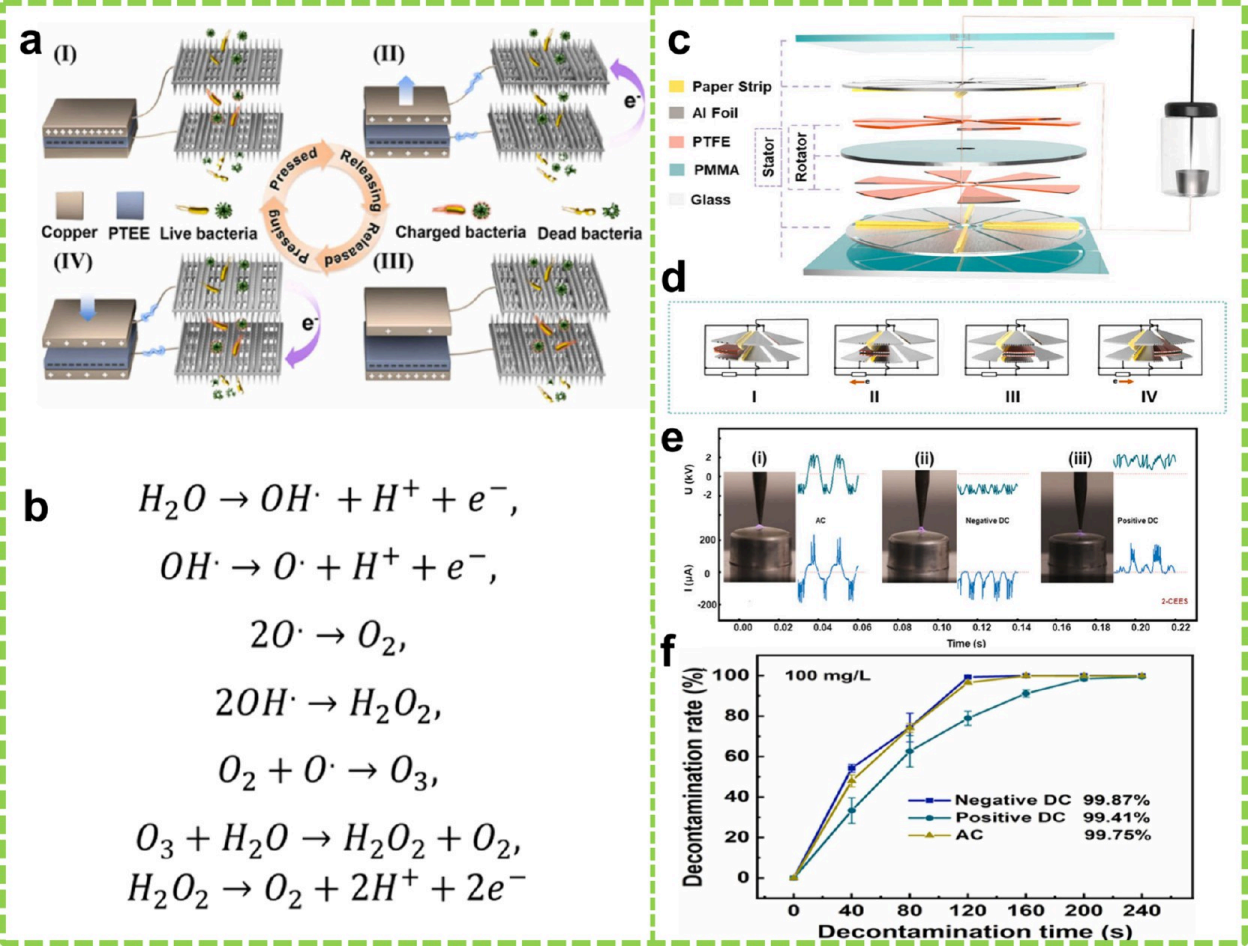
TENG-assisted degradation of medical and chemical warfare hazards. (a) The working mechanism of the TENG for urine neutralization and (b) ROS generation for the degradation of organics present in urine. Reprinted with permission from ref (87). Copyright 2022 Elsevier Ltd. (c) Schematic and the working mechanism of the R-TENG for the decontamination of CWAs, (d) oxidation pathway for 2-CEES, (e) generation of air-plasma, and (f) decontamination rate of 2-CEES with respect to AC, negative DC, and positive DC plasma treatments. Reprinted with permission from ref (170). Copyright 2022 Elsevier Ltd.

Zhang et al. utilized a CS-TENG to decontaminate bacteria and degrade organic compounds in urine using a pulsed SPEC.^87^ The TENG consisted of a PTFE negative frictional layer with a Cu inductive electrode and Cu tribo-electrode as the positive frictional layer attached to acrylic substrates. A sponge layer was placed between the triboelectrode and the acrylic substrate to ensure proper contact between the frictional layers (Figure 15a). Here, cyclic CS between the frictional layers was made easier by using elastic Kapton tape. Two meshes of Cu oxide NWs were stacked, separated by insulating plastic, and rolled into a hollow cylinder. Afterward, electrical connections were made between the TENG and the hollow cylinder. Such a design enhanced the electric field (108 V/m) and improved the bacteria’s inactivation via electrophoresis when the TENG supplied the necessary energy. The generated radical oxygen species have the potential to break down the organic components in synthetic urine (Figure 15b). After treatment with the TENG for 30 min, the reduction rates of the total organic carbon and total nitrogen in the urine reached 30.41% and 33.79%, respectively. After 30 min of treatment with the TENG, the reduction rates for total organic carbon and total nitrogen in the urine were 30.41% and 33.79%, respectively. The degradation rates for specific compounds were 37.39% for urea, 30.50% for uric acid, and 6.25% for creatinine.

#### 4.1.6 Chemical Warfare Agents (CWAs)

Chemical warfare agents (CWAs) are toxic substances designed to harm living organisms, including humans, plants, and animals. Despite their restricted use, CWAs remain a significant public concern due to potential threats from criminal activities and accidents. They can enter the environment through accidental industrial discharges, improper disposal, storage facility leaks, or intentional releases during conflicts.^171^

Sulfur mustard is a well-known CWAs due to its toxicity and low manufacturing cost. Traditional decontamination methods, such as pyrolysis, hydrolysis, oxidation, physical adsorption, and chemical adsorption, often face challenges such as inefficiency, secondary pollution, and high costs. Recent research has explored air plasma technology for decontaminating sulfur mustard surrogates such as 2-chloroethyl ethyl sulfide (2-CEES) and 2-chloroethyl phenyl sulfide (2-CEPS). However, these technologies generally require bulky devices and external power sources, which limit their efficiency. Bai et al. demonstrated an innovative approach using a micro air plasma generated by a self-powered high-voltage device known as a double-layer paper-strip R-TENG (dps-rTENG) for decontaminating 2-CEES.^170^ The dps-rTENG consists of two stators and one rotor mounted on acrylic substrates. Radially arranged Al foil sectors serve as electrodes, and six PTFE film sectors are attached to each rotor side. Paper strips are pasted uniformly between the Al electrodes in ridge shapes, creating a high electric field (3 kV) through friction between PTFE and paper segments that induces a high charge on the Al electrodes (Figure 15c and d). The experiments revealed a 99% removal efficiency of 2-CEES in just 2 min at a 100 mg L^–1^ (40 μL) concentration. The system’s design allows effective control to avoid secondary contamination. Furthermore, experiments using alternating current (AC), negative direct current (DC), and positive DC showed removal efficiencies of 99.87%, 96.56%, and 99.41% in 2, 2, and 4 min, respectively (Figure 15e and f). The superior performance of AC and negative DC over positive DC is attributed to the generation of energetic electrons and active species in the air plasma.

### 4.2 Degradation of VOCs and Other Toxic Chemical Compounds

VOCs are harmful chemical substances that readily vaporize at room temperature, contributing significantly to air pollution. Major sources of VOCs typically include industrial emissions, transportation, and various indoor and outdoor activities.^107^ Compounds of carbonyl and aromatic origin, such as formaldehyde, benzene, toluene, and xylene, are common VOCs, which have been classified as toxic pollutants.^172^ VOCs have both long- and short-term effects on human health and the environment. VOCs have been identified as carcinogens that pose a risk to human health. In many instances, exposure to these compounds can lead to reduced lung function and asthma.^173^ Additionally, VOCs contribute to environmental damage by depleting the ozone layer in the stratosphere, thereby exacerbating the risks associated with the greenhouse effect.^174^ Hence, it is essential to eliminate VOCs from indoor environments or at the very least degrade them to environmentally safe levels.

Phenol is a highly toxic chemical with severe effects on human health, including skin irritation, eye damage, and respiratory issues. Chronic exposure can lead to liver, kidney, heart, and nervous system damage, resulting in serious conditions, such as seizures and coma. Phenol is commonly found in surface water due to its discharge from chemical plants, pharmaceutical companies, and petroleum refineries.^175−177^ Detection and degradation of phenol typically require complex, costly, and energy-intensive equipment.^30,128^ Li et al. introduced a self-powered phenol detection and EC degradation process powered by a WR-TENG.^9^ β-Cyclodextrin (β-CD), a cyclic oligosaccharide composed of α-d-glucose units linked by 1,4-glycosidic bonds, was used to enhance triboelectrification and detect phenol molecules. β-CD has a unique molecular structure with an internal hydrophobic cavity and an external hydrophilic surface.

**Figure 16:**
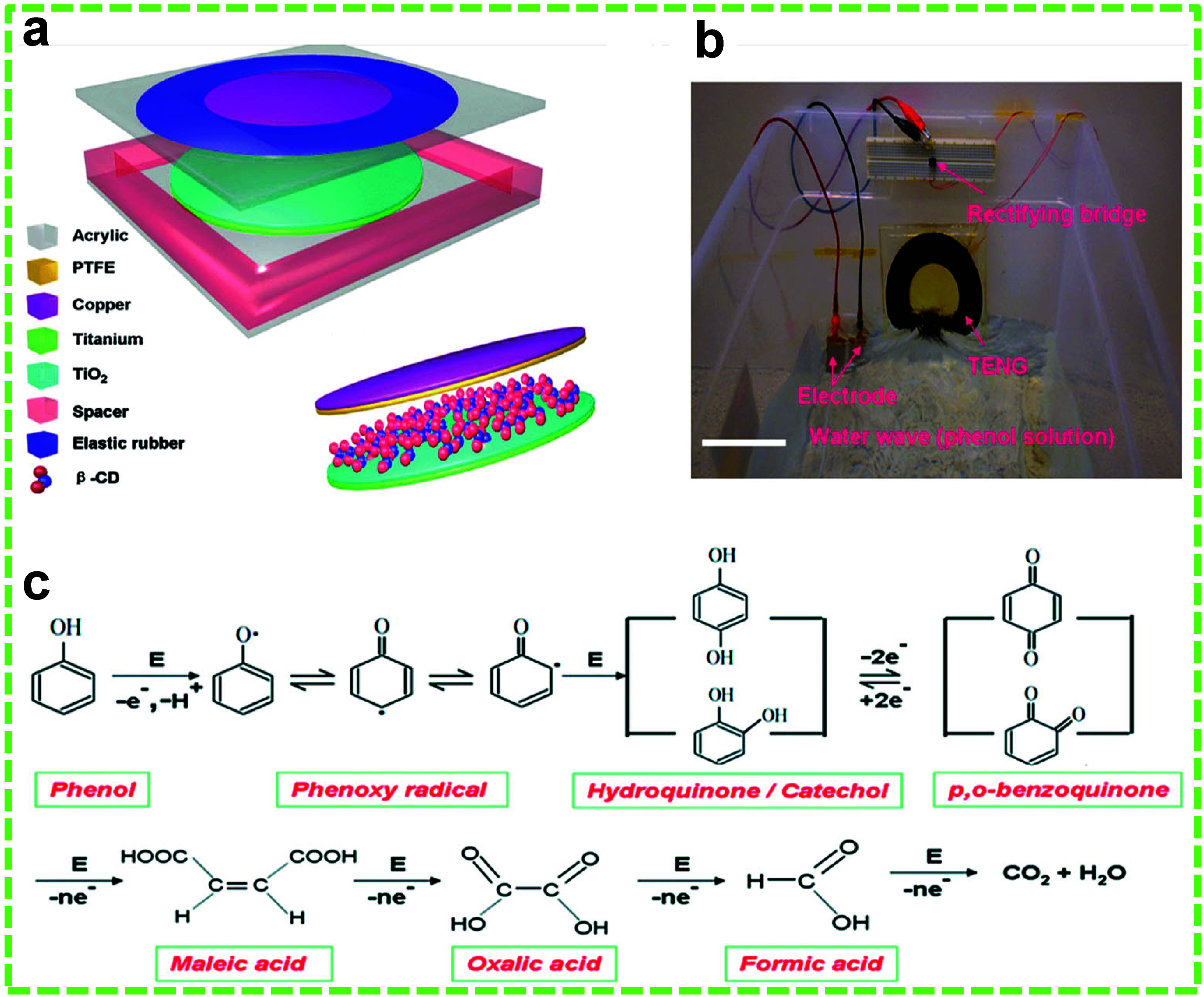
TENG-assisted degradation of phenol. (a) Schematic of the β-CD-added WR-TENG for phenol degradation, (b) the EC system for phenol degradation with β-CD-enhanced WR-TENG, and (c) the proposed stepwise degradation mechanism of phenol. Reprinted with permission from ref (9). Copyright 2015 The Royal Society of Chemistry.

This configuration allows β-CD to function as a host for numerous guest molecules with the appropriate polarity and dimension. The cavity size in β-CD was found to be suitable for phenol adsorption. The designs of the WR-TENG and the SPECs are shown in Figure 16a and b. Briefly, PTFE and TiO_2_ nanowires (NWs) served as the frictional layers, with a Cu-coated electrode for PTFE and a Ti film, where the TiO_2_ friction layer was grown, serving as the other electrode. β-CD molecules were assembled onto TiO_2_ NWs by physical adsorption or H-bonding interactions; additionally, a charge-transfer complex is formed via the coordination effect between the ligand and Ti metal in TiO_2_ under UV irradiation. The degradation process can be outlined as follows: Hydroxyl radicals (•OH) generated at the anode can degrade phenol, with additional degradation occurring at the anode surface. The initial conversion of phenol into a phenoxy radical leads to the formation of hydroquinone and catechol, as •OH radicals attack the benzene ring of the phenoxy radical. These compounds are then degraded into benzoquinone, causing the previously colorless solution to turn yellow. Further benzene ring degradation results in the production of various carboxylic acids, including maleic, oxalic, and formic acids (Figure 16c). These organic acids are mineralized to CO_2_ and water in the subsequent step, resulting in a new, colorless solution. The results showed that degradation performance increased with high wave velocity and β-CD concentration and decreased with increasing phenol content. The device removed 90% of the phenol from the wastewater in 320 min at a wave velocity of 1.4 m s^–1^ and a phenol content of 80 mg L^–1^.

**Figure 17:**
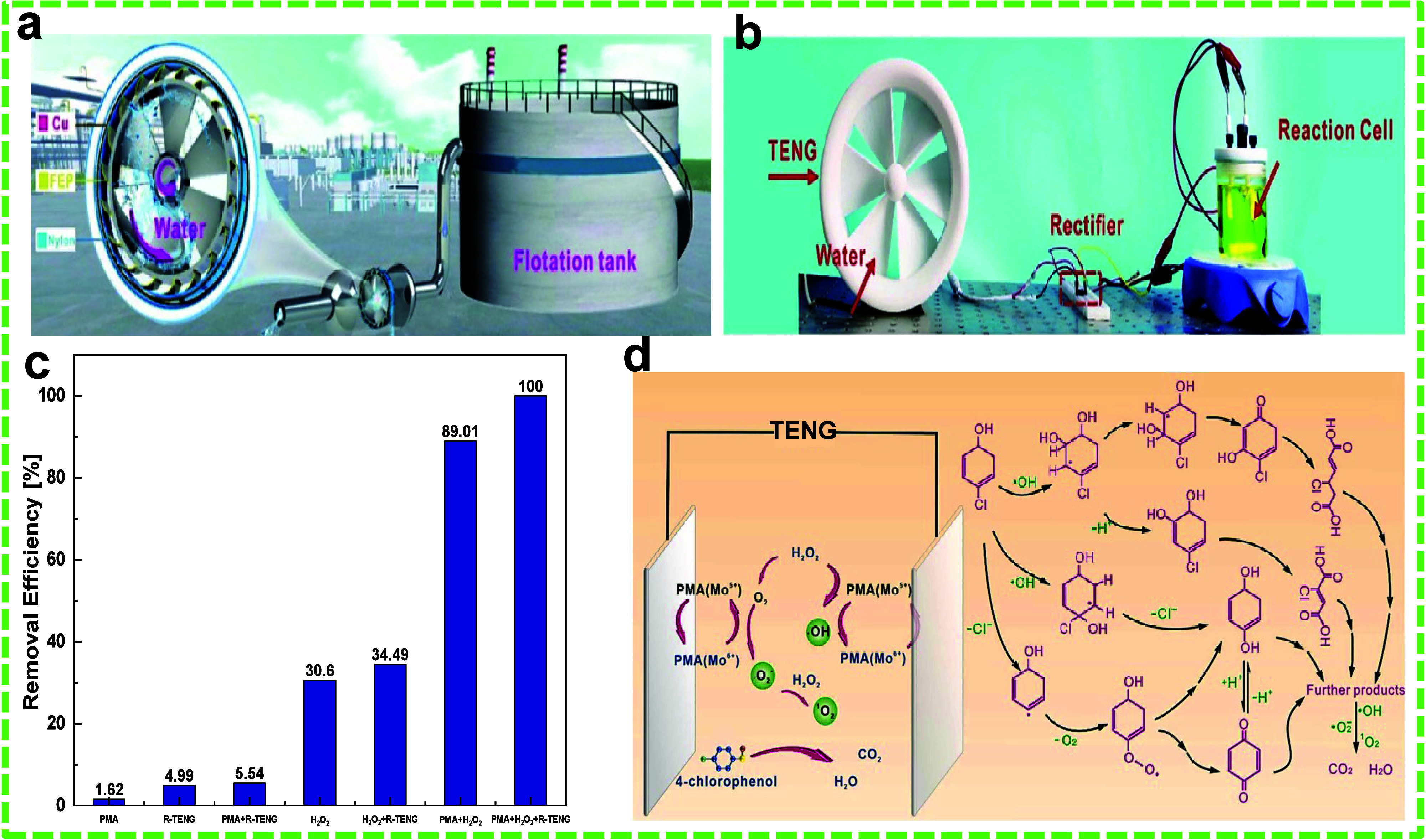
TENG-assisted degradation of 4-CP. (a) Schematic of the WR-TENG system for 4-CP degradation. (b) SPECs for 4-CP degradation driven by WR-TENG. (c, d) Suggested degradation mechanism of 4-CP. Reprinted with permission from ref (116). Copyright 2022 Elsevier B.V.

4-Chlorophenol (4-CP) is a readily water-soluble, colorless, and highly toxic organic pollutant released into the environment from the pulp bleaching process. Using an FS multiunit WR-TENG, Liu et al. demonstrated an environmentally friendly and sustainable alternative for degrading 4-CP.^116^ In this context, perfluoro ethylene propylene (FEP) and nylon function as the sliding and fixed frictional layers, respectively, for each R-TENG unit. Concurrently, Cu foil was utilized as the complementary electrode, as depicted in Figure 17a. Water-resistant glue was used to seal the TENG units for protection. The electrochemical system consisted of a Pt cathode and a glassy carbon electrode, while phosphomolybdic acid (PMA; H_3_PM_o12_O_40_) served as the catalyst (Figure 17b). The degradation experiment was carried out in a 100 mL solution containing 0.78 mM 4-CP, 3.9 mM PMA, and 19.5 mM H_2_O_2_ at 90 °C. When radical species like singlet oxygen, O_2_^–^, and •OH radicals are produced, 4-CP is degraded and mineralized into CO_2_ and H_2_O. In this study, the degradation efficiency was found to be 100% in 120 min when a rectified DC output was provided by the WR-TENG (Figure 17c and d).

**Figure 18:**
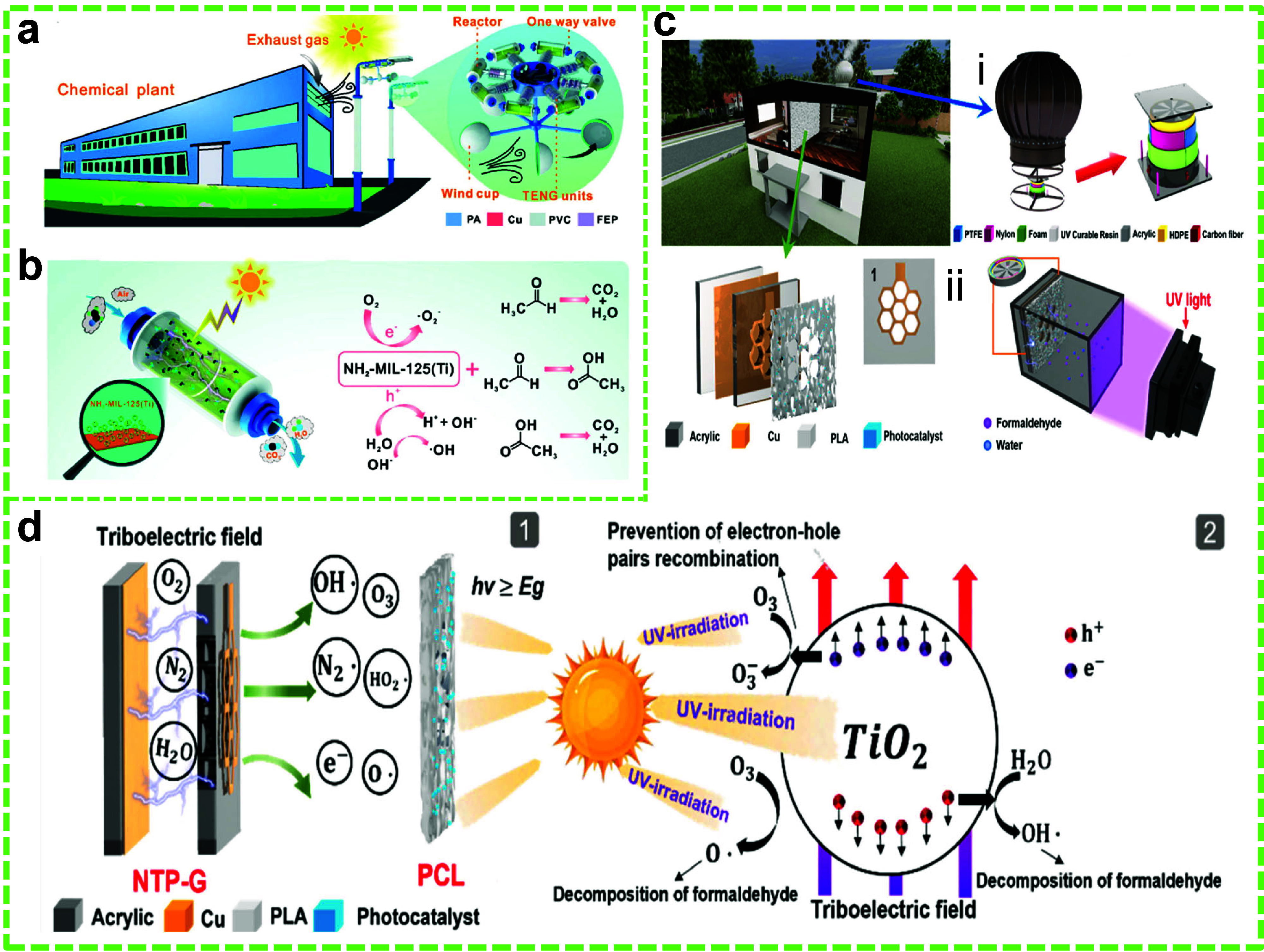
TENG-assisted degradation of acetaldehyde and formaldehyde. (a) Schematic of the application scenario and PWD-TENG construction and (b) the degradation mechanism of acetaldehyde by PWD-TENG and NH_2_-MIL-125(Ti) photocatalyst. Reprinted with permission from ref (178). Copyright 2021 American Chemical Society. (c) Schematic of the application scenario and R-TENG construction for formaldehyde degradation and (d) NTP-G, PCP, and R-TENG-based degradation mechanism of formaldehyde. Reprinted with permission from ref (80). Copyright 2022 Elsevier Ltd.

The carcinogenic acetaldehyde, released from various sources including industrial production, automobile interiors, exhaust, furniture, and cigarettes, harms the environment and the human body. Fu et al. developed a piston-like WD-TENG (PWD-TENG) for acetaldehyde adsorption and PEC degradation.^178^ As shown in Figure 18a, the PWD-TENG had eight gas chambers with photoreactors and one-way gas control valves that were radially spaced at 45° intervals on an eccentric wheel. The photoreactor contained two conductive substrates, each coated with 5 mg of NH_2_-MIL-125(Ti) photocatalyst NPs. In the degradation mechanism, the TENG units generated electrostatic fields that assisted in absorbing and later degrading acetaldehyde into H_2_O and CO_2_ when exposed to light (Figure 18b). The electrodes used in the TENG units were copper foils, and the positive and negative friction layers were made of polyamide (PA) and FEP films. In 30 min, the system achieved 63% removal efficiency when operated under 3 m s^–1^ artificial wind and from 280 ppm initial concentration, but the performance of the PEC degradation system declined over successive cycles.

In a more recent study, Zheng et al. developed an R-TENG based on contact electrification and the air breakdown effect to provide the necessary electric field for the degradation of formaldehyde.^80^ In this case, the degradation of the formaldehyde was accomplished through a synergistic action of a nonthermal plasma generator (NTP-G), TiO_2_ photocatalyst, and electric field supplied by the R-TENG (Figure 18c(i)). In the R-TENG, an HDPE frictional layer was attached to the rotor, while the PTFE and nylon frictional layers were alternately attached to the stator as the other two frictional layers. The charge collectors were microdiameter carbon fiber electrodes placed between the PTFE and nylon layers. The frictional layers’ electron affinity is in the following order: PTFE > HDPE > nylon. When HDPE contacts PTFE in a clockwise direction, it becomes positively charged, while PTFE becomes negatively charged. As nylon encounters positively charged HDFE, HDPE becomes negative, and nylon becomes positive. When HDPE with different charge polarities comes close to the collector electrodes, a strong electric field causes air breakdown and generates a conducting path. Electrons can, therefore, move between the collector electrodes via external circuitry from the alternatively charged HDPE layer.

A continuous DC output is produced if the rotor spins clockwise. NTP-G was built using a flat Cu electrode on an acrylic substrate and a honeycomb Cu electrode on a different acrylic substrate (i.e., an air breakdown layer). For the generation of NTP and O_3_, the NTP-G was connected to the TENG. PLA was used to print a porous 3D framework, which was then functionalized with a TiO_2_ photocatalyst and laminated onto NTP-G for photocatalytic degradation (Figure 18c(ii)). Ozone functions as an electron donor and inhibits the recombination of electrons and holes. Additionally, the electric field provided by the TENG further inhibits the recombination of the electron and hole pairs (Figure 18d). All three parts of the degradation system work in synergy, and the experiment showed that it could remove 94% formaldehyde enclosed in a 13 L closed chamber within 10 min. The benefit of the system is that it can perform individually without depending on light energy all the time.

Table 1 collects some of the recent works where a TENG was associated with the degradation mechanism of organic pollutants.

**Table 1:** TENG-Assisted Degradation of Organic Compounds

	TENG material							
sl.	frictional layers	electrodes	compounds degraded	degradation performance	TENG mode	driving force	TENG type	ref
1	PTFE and Al	Al and Cu	MO	76% in 120 min	CS	mechanical energy	stacked	(84)
2	PDMS and PET	ITO	MO		CS	mechanical energy	stacked	(2)
3	PTFE and nylon	Cu	MO		CS	mechanical energy	stacked	(141)
4	PTFE and Cu foils	Cu	MO, MB, MG	88.9% MO, 91.7% MB, and 94.1% MG in 150 min	CS	mechanical energy	vortex-like	(142)
5	Kapton and Cu	Cu	RhB	close to 100% in 15 min	FS	mechanical energy of water	rotational	(40)
6	Kapton and Cu	Cu	RhB	87.5% in 120 min	FS	mechanical energy of wind	rotational	(143)
7.	Kapton and Al	Al	RhB and formaldehyde	50% reduction in degradation of RhB and 2 times enhancement in formaldehyde degradation	SE	mechanical energy	stacked	(13)
8	La_0.05_ Fe_0.05_ codoped ZnO nanoarrays and PDMS	Zn and Cu	RhB	100% in 5 h	CS	mechanical energy	stacked	(88)
9	PTFE and Cu	Cu	MR	almost 100% MR in 160 min	CS	mechanical energy	stacked	(83)
10	Kapton and Al	Al	azo dye (4-aminoazobenzene) and 2-(4-dimethylaminophenylazo)benzoic acid	99.9% in 12 min and 98.4% after 600 min	FS	mechanical energy	rotational	(10)
11	PTFE and Al	Al	MY and Basic Orange- 2	99% MY in 120 min and 98% Basic orange- 2 in 2 min	CS	mechanical energy	stacked	(132)
12	PTFE and Cu	Cu	MB	97% in 140 min	CS	mechanical energy	stacked	(131)
13	PTFE and Al	Al	MB	98.1% in 58 min	CS	mechanical energy	stacked	(129)
14	PTFE and Al	Al	crystal violet and orange IV	96.0% crystal violet in 60 min and 95.4% Orange IV in 60 min	CS	mechanical energy	stacked	(130)
15	PTFE and Cu	Cu	BG DG	88.26% BG in 40 min and 89.6% DG in 1.5 h	FS	mechanical energy	rotational	(145)
16	PTFE and TiO_2_ NWs	Cu and Ti	Phenol	90% in 320 min	CS	mechanical energy of water	stacked	(9)
17	FEP and nylon	Cu	4-CP	100% in 120 min	FS	mechanical energy of water	rotational	(116)
18	PA and FEP	Cu	acetaldehyde	63% in 30 min	CS	mechanical energy of wind	rotational: radial engine	(178)
19	HDPE, PTFE, and nylon	carbon fiber	formaldehyde	94% in 10 min	FS	mechanical energy of wind or thermal convection between indoor or outdoor air	rotational	(80)
20	FEP and Cu	Cu	gummy materials of ramie fibers	90% of the pollutants in the wastewater	FS	mechanical energy of water	rotational	(82)
21	Kapton and Acrylic	fabric electrode	levofloxacin	86.6%	FS	mechanical energy of wind	rotational	(154)
22	cellulose and FEP	Cu	tetracycline	92.72% in 60 min	CS	mechanical energy of water	corona-shaped	(155)
23	nylon and FEP	Cu	ATZ	91.47% in 30 min	CS	mechanical energy of water	piston	(163)
24	PTFE and Cu	Cu	urea, uric acid, and creatinine	37.39% urea, 30.50% uric acid, and 6.25% creatinine in 30 min	CS	mechanical energy	stacked	(87)
25	PTFE and paper	Al	2-CEES	99% in 2 min	FS	mechanical energy	rotational	(170)

### 4.3 Degradation of Inorganic Gases

When fossil fuels are burned, exhaust gases and other contaminants are produced and released into the atmosphere, causing air pollution.^179,180^ Many of the pollutants are inorganic, originating from industrial fumes, transport emissions, power plants, and natural incidents like volcano eruptions.^109^ Major inorganic gaseous pollutants include carbon monoxide (CO), carbon dioxide (CO_2_), sulfur dioxide (SO_2_), nitrous oxides (NO_*x*_), hydrogen sulfide (H_2_S), and hydrogen fluoride (HF). Long-term exposure to air contaminated with these pollutants can cause health problems, such as lung cancer, cardiopulmonary mortality, and respiratory illnesses. For instance, PM_2.5_ formed from SO_2_ and NO_*x*_ can cause respiratory and cardiac problems.^16,107,14^ Additionally, air pollution leads to environmental consequences, like the formation of smog and acid rain, which can damage the ecosystem, crops, and visibility. It is important to find practical and affordable solutions to enhance people’s quality of life.^181−183^ Utilization of TENGs for self-powered electrochemical removal of SO_2_,^3^ NO_*x*_,^69^ and CO_2_^117^ has already been researched to remove hazardous gases from the air.

**Figure 19:**
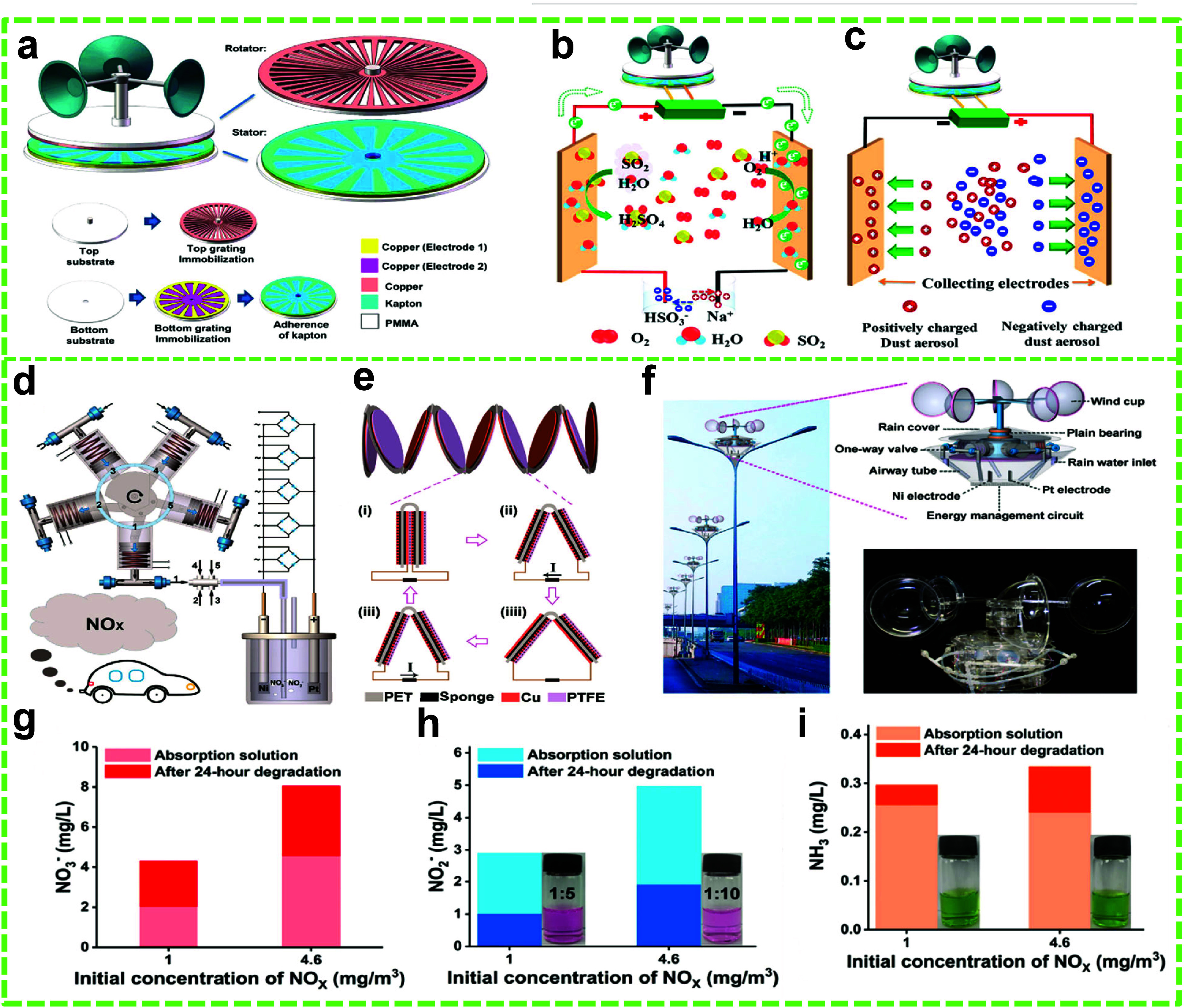
TENG-assisted removal of inorganic gases. (a) Schematic of the WD-RTENG for SO_2_ removal. (b, c) The mechanism of SO_2_ and dust particle removal by WD-RTENG. Reprinted with permission from ref (3). Copyright 2015 Elsevier Ltd. All rights reserved. (d, e) Schematic and working mechanism of the engine-like CS-TENG. (f) Illustration of the wind-driven mechanism of the CS-TENG for NO_*x*_ removal. (g) Detection results of NO_3_^–^, (h) NO_2_^–^, and (i) NH_3_ after 24 h of self-powered degradation using a 20 mL absorption solution from the simulation absorption experiment. Reprinted with permission from ref (69). Copyright 2020 American Chemical Society.

The removal of SO_2_ has been accomplished using metal-catalyzed and electrocatalytic oxidation processes.^110,111^ However, metal-catalyzed oxidation depends on novel metals, which are expensive and scarce, while electrocatalytic oxidation requires external power. With a WDR-TENG, Chen et al. demonstrated the EC removal of SO_2_; additionally, the system can electrostatically adsorb dust particles.^3^ The stator and rotor of the R-TENG were built with poly(methyl methacrylate) (PMMA) substrates, Kapton film, and grated Cu electrodes arranged as shown in Figure 19a. The rotor was equipped with three wind cups that were intended to be propelled by the wind. The R-TENG operated at 300 V, a voltage safe from the generation of ozone and other unwanted byproducts. The oxidation mechanism of SO_2_ into H_2_SO_4_ proceeds as illustrated in Figure 19b and c. Briefly, at the anode, H_2_SO_4_ is produced when SO_2_ loses two electrons; at the cathode, O_2_ gains those electrons to produce H_2_O. The self-powered system is economically convenient and straightforward in design and uses readily available materials.

Acid rain can harm crops and other plant life, and NO_*x*_ is a significant contributor to this problem.^184,185^ In humans, it can cause chronic diseases and irritate the respiratory tract.^186,187^ The current methods for removing NO_*x*_ involve catalytic reduction, which concentrates on the primary sources of pollution and uses NH_3_ and urea as the reducing agents, making the process impractical in an outdoor setting. Due to NO_*x*_’s low solubility in water, other techniques, including pressurization and high-capture performance materials, were investigated to capture a trace amount of NO_*x*_, which inevitably increases equipment and material requirements.^188−190^ In light of this, Han et al. developed a NO_*x*_ adsorption and degradation system using wind-driven stacked CS-TENG units in a radial engine-like structure (Figure 19d–f). Each stacked TENG unit was operated by using piston assemblies and was connected to individual gas chambers. To allow contaminated air into the system, one-way valves were installed on either side of the gas chamber.
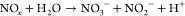
4

The piston movements and one-way valves were controlled to permit contaminated air to enter the system and mix with water. After several water washes, the system was left with harmful nitrate and nitrite substances for degradation (eq 4).

In their study, Li et al. used a stacked TENG configuration consisting of eight individual units to generate sufficient energy for the catalytic system. They chose PET as the substrate material to support the components and ensure continuous bending, while PTFE and Cu films were selected as the frictional layers. The setup included a piston attached to one end of the stacked TENG units and a gas chamber connected to the other end. For the experimental phase, KNO_3_ and KNO_2_ were used as model contaminants. The electrochemical system featured a Ni foam catalyst-based working electrode and a Pt counter electrode, with 0.1 mol L^–1^ K_2_SO_4_ serving as the electrolyte. The following reactions can occur:
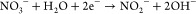
5
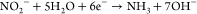
6
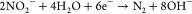
7
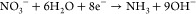
8

In a real-world simulation, radial engine-shaped TENGs were operated at a wind speed of 6 m s^–1^ with a 20 mL absorption solution added to the catalytic system. The system was observed over 24 h. The results were promising, demonstrating the potential for using such equipment for NO_*x*_ absorption and degradation in outdoor environments (Figure 19g–i).

**Figure 20:**
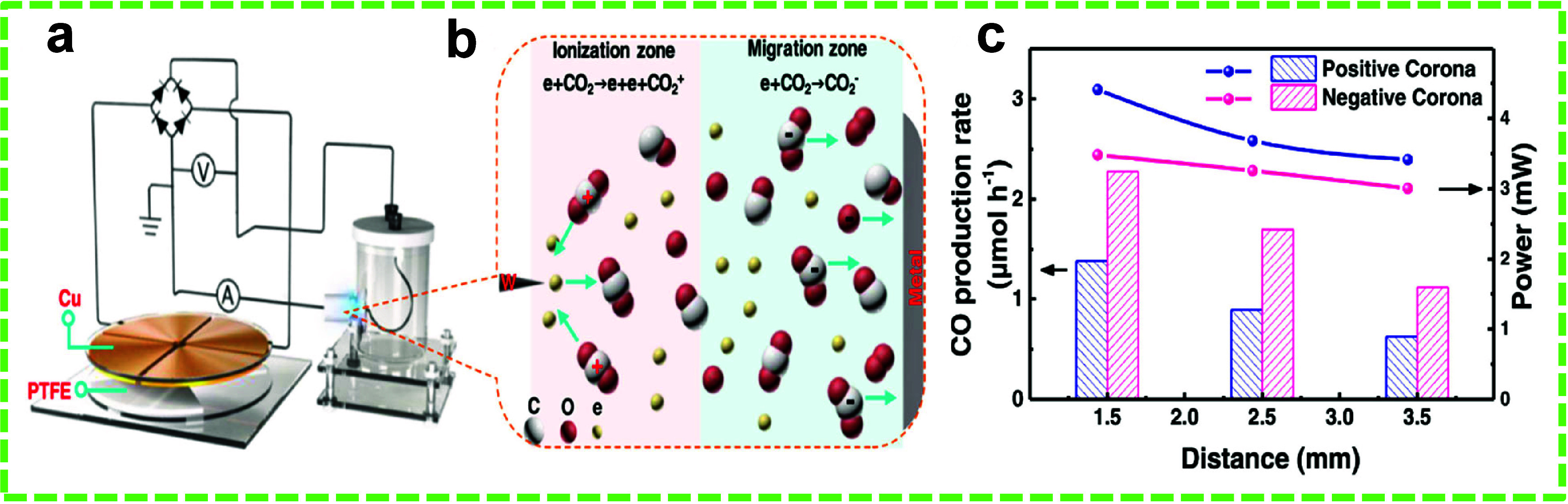
(a) Schematic of the experimental setup for WD-RTENG-assisted decomposition of CO_2_. (b) The ionization and the migration zone. (c) Influence of corona polarity and needle-plate distance on the decomposition of CO_2_ into CO. Reprinted with permission from ref (191). Copyright 2021 Elsevier Ltd.

Compared to the popular high-temperature (>2000 K) decomposition process, the decomposition of CO_2_ into CO at room temperature and atmospheric pressure using a green and sustainable approach is highly desirable. To that end, a plasma decomposition system was developed by Li et al. with a WD-RTENG to sustainable break down highly stable CO_2_ into CO (Figure 20a).^191^ The decomposition system consists of a gas reactor, an electrical test system, and a freestanding TENG made of PTFE and Cu. The rotation of PTFE caused an alternating current to develop, which changed a full-wave rectifying bridge to a direct current. After that, this direct current was connected to needle-plate discharge electrodes inside the gas reactor, which had previously been filled with pure CO_2_; the plasma was generated upon the gas ionization. The ionization zone was deemed near the needle surface with a strong electric field for corona discharge. On the other hand, the migration zone with low electric field strength had no ionization capability and was designated outside the ionization zone (Figure 20b). According to the study, under a needle-plate distance of 1.5 mm, CO_2_ decomposed into CO at rates of 2.2 and 1.3 mol/h for negative corona and positive corona, respectively. The distance between needle-plate electrodes influences the decomposition of CO_2_ and the generation of CO (Figure 20c).

The Review shows that TENGs, constructed from diverse materials and structures, have been extensively researched for the degradation of various organic compounds, VOCs, and inorganic gases. Their primary advantage is their ability to provide a self-powered system for driving degradation mechanisms. Given the prevalence of pollutants in air and water, TENGs are well-suited to harness unused mechanical energy such as airflow, water flow, and other mechanical sources and convert it into electrical energy with high efficiency.^8,192,193^ This eliminates the need to install additional power generation and supply units. Such a TENG-based energy generator is quite inexpensive compared with the installation and operation of other systems. Further, TENGs ability to work under ambient conditions provides an advantage over many other processes like steam turbines, gas turbines, and geothermal power plants, which require high pressure and temperature.^193−195^ Self-generated power can be used to drive photolytic or electrolytic activities. High-voltage electric pulses generated by TENGs have been proven effective in oxidizing inorganic gases, powering plasma reactors, and corona discharge mechanisms, which can play an important role in degrading various pollutants. Further, TENG-driven mechanisms can efficiently avoid forming any byproducts, unlike other processes, like fossil fuel combustion.^204^

## Perspectives and Conclusions

In summary, our environment is fraught with various pollutants that pose a threat to human existence. Among these, organic chemicals, VOCs, and inorganic gases play significant roles. TENG-assisted devices for pollutant degradation show great promise compared to traditional techniques such as oxidation, biotreatment, and enzyme degradation, as well as modern developments such as nanofiltration, electrostatic absorption, activated carbon, and biomass-based filtration, primarily due to their self-powering capabilities. Among the available green-energy-harvesting technologies, TENGs have shown particular potential due to their simple design, low-cost materials, and ability to harvest low-amplitude energies through the electrostatic induction principle. Of the four major principles, CS is known for greater durability but requires more space during operation, impacting packaging. In contrast, while LS offers improved packing, it is relatively short-lived. The SE mechanism provides more flexibility in architecture and construction, allowing energy harvesting from freely moving objects, but lacks energy output. FS-TENGs enable energy harvesting from a wider range of sources by being attachable to rotating configurations and other flowing motions, such as water and air.

This Review specifically examines three categories of chemical pollutants: organic compounds, VOCs, and inorganic gases. Due to their nonbiodegradability and persistent nature, these pollutants persist in the environment and cause harm. While TENGs can effectively eliminate pollutants in particulate matter form using their inherent electrostatic field generation ability, they are insufficient for degrading more complex VOCs and inorganic matter. To address this limitation, photoelectrocatalysis and electrocatalysis can be integrated with TENGs to provide significant support in efficiently addressing these pollutants.

The use of TENGs in conjunction with photocatalysts has proven effective in breaking down toxic organic pollutants and VOCs by expediting charge separation and migration. This leads to a decrease in electron–hole pair recombination. Additionally, TENGs can be utilized for electrochemical degradation through electrolysis, enabling the breakdown of organic components and harmful VOCs by supplying energy through successive transformation and rectification processes. Furthermore, the TENG-enhanced electrostatic absorption effect, driven by the Coulomb force, can be harnessed to accelerate the removal of polar organic pollutants. Radical ions such as OH^–^ and O_2_^–^ are renowned for breaking down a wide range of organic compounds, VOCs, and inorganic gases, such as polysaccharides, phenols, and CO_2_. TENGs can facilitate the generation of these radicals by creating an electric field and influencing the movement of anions and cations. Moreover, TENG-powered EF processes can be coupled with PDC to mitigate the impact of electrode passivation, thereby enhancing the degradation of organic antibiotics. Notably, TENGs have also been found to power contemporary technologies such as electrophoresis, air plasma generation, and plasma degradation, which aid in breaking down organic components, VOCs, and inorganic gases. Furthermore, TENGs can serve as an energy source to support the degradation of various VOCs by fueling the adsorption process using the appropriate host chemicals. It is also worth noting that the harnessed energy from the surroundings can drive the electrochemical processes for inorganic harmful gases like SO_2_, NO_*x*_, and CO_2_ and can also electrostatically absorb matter in dust particle form.

**Figure 21:**
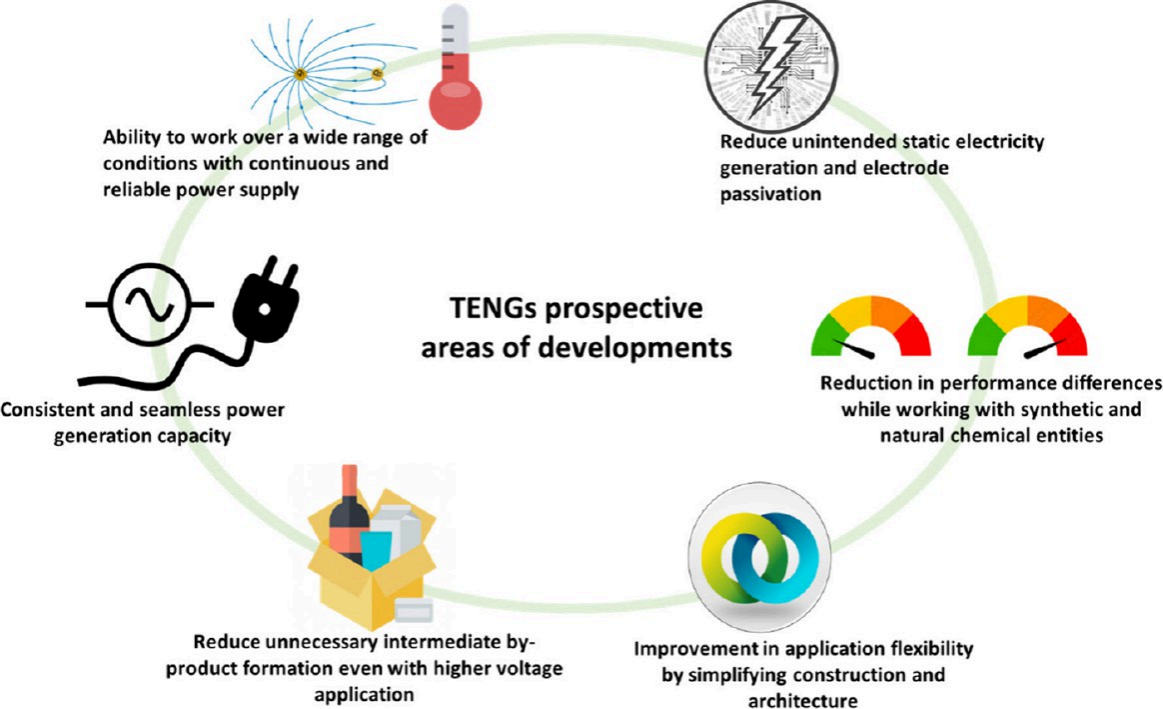
Prospective areas for the development of TENGs applied to the self-powered degradation of chemical pollutants.

Although TENGs have shown incredible promise as an emerging technology for wastewater treatment and toxic chemical degradation, some major challenges still need to be addressed. Figure 21 summarizes the field of developments for TENGs applied to the self-powered degradation of chemical pollutants. One of the greatest advantages of TENGs is their ability to scavenge unused energies from the surroundings to make the device self-powered, eliminating the requirements of any external power source.^2^ However, for the device to perform consistently, the power source is expected to work over a wide range of conditions with a continuous and reliable power supply. For example, the degradation performance of TENGs needs to be observed under conditions like high temperature, magnetic field, electrical field, vacuum conditions, etc.^4^ TENG performance, however, has room for improvement when subjected to such conditions. Therefore, the functionality of a TENG in harsh environments should be the subject of more research studies.

On top of that, TENGs mostly produce energy in a microrange, which might not be sufficient to ensure a consistent supply of power to drive chemical degradation processes like electrochemistry, photochemistry, electrodegradation, and electrocatalysis.^1,120^ Scientists are investigating ways to improve the power generation capacity by optimizing structural designs, constituent materials, and chemical functionalization.^196−198^ Simultaneously, structural designs are being optimized to improve degradation performance.^4^ Nevertheless, the output performance is still not satisfactory and requires further exploration.

To improve the energy output of TENGs, a common practice is to use hybrid structures, in which TENGs are combined with other common NGs such as PENGs, PyNGs, and TEGs.^199^ However, in addition to making the design more complex, this reduces the application flexibility and increases manufacturing costs. Furthermore, it has been reported in some cases that the total energy output of the hybrid structure is weakened rather than improved due to AC output in nature. Numerous research papers have also addressed this issue, which involved using a rectifier by integrating a full wave bridge circuitry system to convert AC output to the DC form.^2^

Among the other issues associated with TENGs for chemical degradation applications is unintended static electricity generation, which can result in ignition, electric shocks, and dust explosion.^200^ Electrode passivation is another common issue associated with the charge accumulation of TENGs, which can hinder the chemical removal efficiency of the whole device. This commonly occurs on the electrode surface. For metal electrodes, non-negligible charge polarization results in an open circuit voltage difference within the TENG system.^201^ To help with the situation, researchers have proposed using pulsed direct current (PDC). However, integrating a full PDC to TENG has turned out to be quite challenging, as a superimposition of multiple electrodes can create problems. During the TENGs design phase, optimization of the angle ratio of the electrodes may be investigated to resolve this problem.^154^ Inconsistent and uneven energy output in the form of a high-voltage pulse is problematic because the entire system may become unsettled.^141^ A power management system with TENGs has been recommended to address this limitation, which usually involves installing a Li-ion battery to store the generated power. This concern about constant power supply has led researchers to prefer using a storage device with TENGs rather than directly powering the electro/photodegradation. Nevertheless, this has compromised the simplicity of the device.^2^ Chemical degradation performance was found to be improved with the integration of power management. Feng et al., for example, demonstrated that integrating a power management unit increased electrochemical cell performance by 3.5×.^202^ Another method of controlling pulse intensity is incorporating additional components such as capacitors, circuit bridges, etc.;^141^ however, these will make the device complex and increase the cost. Hence, future research should concentrate on power management for TENG-driven effective chemical degradation performance.^4^

The inability of a TENG to be integrated separately in situations where electrostatic absorption is not practical is one of the main issues with TENG-based chemical degradation devices. For example, a TENG alone cannot degrade VOCs and needs to be combined with photoelectrocatalysis or electrocatalysis. Integration of a TENG with photo- or electrodegradation systems has improved the degradation performance and reduced the degradation time significantly. However, the performance has yet to reach its peak. For example, Su et al.’s study showed the degradation of MO from wastewater could only reach up to 76%.^4^ Another study with MO demonstrated 80% degradation in 144 h.^2^ Therefore, the authors recommend further research to improve the degradation rate and the duration of performance.

Using a higher selective voltage to degrade chemicals can result in intermediate byproducts, sediments, and so on. Insoluble intermediate products, for example, are produced when the MO is degraded using a TENG-assisted device. The same is true for higher chemical concentrations, which also produce byproducts.^2,203^ Future studies can focus on how to reduce the generation of undesirable byproducts. Currently, CS and FS-TENGs are more often used for SPECs. However, if not properly designed, sometimes installing CS-TENGs into chemical degradation systems becomes challenging due to structural constraints.^142^

TENG-integrated chemical degradation systems show much better performance (>80%) in the case of synthetic dyes and chemicals. However, performance is still not at the same level in the case of organic pollutants like antibacterials, pesticides, and urine components. Furthermore, for antibiotic removal, the acidic condition shows better results, as in the alkaline condition −OH release is reduced.^154^ Future studies may be directed to improve the performance for organic pollutants and alkaline conditions.

To sum up, this Review is focused on the application of TENGs for chemical degradation. Chemical degradation is significant for wastewater and toxic air treatment to prevent environmental degradation. TENG-based SPEC is already a suitable and promising solution, in comparison with energy-consuming electrochemical solutions and filtering mechanisms. Lower energy demand and flexibility with structural design help to adapt the device to various situations. A wide range of common organic and inorganic chemical pollutants in liquid and gaseous forms can be treated satisfactorily with TENG-assisted devices. Despite some existing challenges, TENGs provide overall solutions for enormous power demand, lack of performance, design complexity, and higher cost, associated with other chemical degradation mechanisms. Thus, the self-powered TENG-assisted device’s contribution to wastewater treatment and pollution degradation is promising for building a sustainable green technology-driven future.
